# Edible Oil-Based Extraction of *Cannabis sativa* L. Roots: Effect of Solvent and Temperature on Friedelin Yield and Antioxidant Activity

**DOI:** 10.3390/molecules31091473

**Published:** 2026-04-29

**Authors:** Johana Angelica Guerrero Amaya, William Z. Xu, Paul A. Charpentier

**Affiliations:** Department of Chemical and Biochemical Engineering, Faculty of Engineering, University of Western Ontario, London, ON N6A 5B9, Canada; jguerre7@uwo.ca (J.A.G.A.); william.z.xu@hotmail.com (W.Z.X.)

**Keywords:** *Cannabis sativa* L. roots, Friedelin, edible oil-based extraction, hemp seed oil, MCT coconut oil, grape seed oil, DPPH assay, ABTS test, β-carotene bleaching method, ANOVA

## Abstract

The roots of *Cannabis sativa* L., historically overlooked, are gaining attention as a potential source of bioactive compounds with antioxidant, antimicrobial, and anti-inflammatory properties. While previous studies have focused on extractions using ethanol, water, or supercritical CO_2_, the feasibility of edible oil-based extraction remains largely unexplored. This study evaluated the extraction of root compounds using hemp seed oil, MCT coconut oil, and grape seed oil at six temperatures (50–90 °C). Extracts were analyzed by GC–MS for compound identification and quantification, and antioxidant activity was assessed using the DPPH assay, ABTS test and β-carotene bleaching method, with results statistically evaluated by ANOVA. Friedelin was successfully extracted with all oils, with grape seed oil yielding the highest concentration (0.810 mg/g dry roots), achieving recoveries higher than those previously reported for ethanol-based extractions. All extracts demonstrated positive antioxidant activity, with grape seed oil, both alone and combined with extracts, showing higher values across the three methods. ANOVA revealed a significant effect of solvent type on both Friedelin concentration and antioxidant capacity. These results demonstrate that edible oils are effective solvents for extracting bioactive compounds from *C. sativa* roots, supporting their potential application in cosmetic or medicinal formulations.

## 1. Introduction

Recent concerns regarding the rise in antibiotic and antifungal resistance, the effects of photoaging and uncontrolled oxidative stress on skin tissue, and the adverse side effects associated with conventional anti-inflammatory drugs have intensified the search for sustainable and biologically derived alternatives with antimicrobial, antioxidant, and anti-inflammatory properties. Plant-based extracts offer a promising solution, as plants are renewable resources rich in bioactive compounds that may address these health challenges [1].

Hemp *Cannabis sativa* L. is an herbaceous plant belonging to the Cannabaceae family, native to Central Asia [1,2]. Historically, it has been used in traditional medicine to treat conditions such as gout, arthritis, fever, inflammation, skin burns, and infections, and it has also served as a source of textile fiber [3]. Although cannabis roots were long overlooked and discarded, they are now receiving growing attention in medical research due to their rich composition of phytochemicals, cellulosic and woody fibers, and the potent bioactivities of their metabolites on human health [2]. Consequently, the unique chemical profile of cannabis roots suggests their potential in addressing contemporary health concerns.

The potential applications of cannabis roots and their extracts depend on the specific compounds they contain. *Cannabis sativa* roots contain a variety of active compounds, including triterpenoids, monoterpenes, alkaloids, and sterols [3]. Specifically, these plant roots are a source of phytochemicals such as Friedelin, Epifriedelanol, Carvone, Dihydrocarvone, Cannabisativine, Anhydrocannabisativine, Sitosterol, Campesterol, Stigmasterol, and N-(p-hydroxy-β-phenylethyl)-p-hydroxy-trans-cinnamamide [3]. Among these, Friedelin and Carvone are of particular interest. Friedelin, or friedelan-3-one (C_30_H_50_O), the most abundant triterpenoid in cannabis [4], is a pentacyclic triterpene ketone with demonstrated antimicrobial [5], antitumor [6], anti-inflammatory [7], analgesic [7], antipyretic [7] and antioxidant activities [8]. Carvone (C_10_H_14_O), a monoterpenoid ketone, has been reported to exhibit diverse biological activities, including significant antimicrobial and antifungal properties [9], high antioxidant activity [10], and anti-inflammatory effects [11].

Given these properties, recent research has increasingly focused on the antioxidant capacity of *Cannabis sativa* L. root extracts. Kornpointner et al. [12] identified 20 secondary metabolites using ethanol and supercritical CO_2_ extraction (SFE-CO_2_), assessing antioxidant activity through ABTS (2,2′-azino-bis(3-ethylbenzothiazoline-6-sulfonic acid)), FRAP (Ferric reducing antioxidant power), and cellular assays. Similarly, Giselle et al. [13] investigated the antioxidant activity of ethanol and water-based root extracts using ABTS tests, finding that although root extracts showed lower antioxidant activity than inflorescence extracts, the activity remained significant. More recently, Gagné et al. [14] performed extractions using water, ethanol, and acid–base solvents, identified compounds by UPLC, and assessed antioxidant activity through free radical scavenging (ABTS), metal chelation, and lipid peroxidation inhibition assays.

While these studies assessed antioxidant activity using radical scavenging assays, specifically single electron transfer (SET)-based methods like ABTS [15,16], the DPPH (2, 2-diphenyl-1-picrylhydrazyl) assay is also a widely recognized SET method [17,18]. Notably, Friedelin isolated from other plant species has demonstrated high scavenging effects specifically in DPPH assays [8]. Complementary to these SET approaches, hydrogen atom transfer (HAT)–based methods such as the β-carotene bleaching assay have been broadly employed to evaluate the antioxidant capacity of plant extracts [16,19], though this assay has not been specifically applied to cannabis root extracts.

The studies mentioned above have focused on extraction using ethanol, SFE-CO_2_ or water-based solvents. While SFE-CO_2_ is a non-polar technique that can produce oil-like extracts, the potential of using liquid edible oils as the primary extraction solvents for *C. sativa* roots remains unexplored. In this context, hemp seed oil, a derivative of *Cannabis* spp. (hemp), is specifically exempt from the Cannabis Act under Canada’s Industrial Hemp Regulations [20] and is permitted for use in cosmetic formulations [21]. This regulatory status, together with the unexplored potential of edible oil-based extraction, highlights the suitability of hemp seed oil as a direct extraction medium and carrier for bioactive compounds, eliminating the need for solvent separation. To evaluate its performance, hemp seed oil can be compared with other commonly used cosmetic carrier oils, such as MCT coconut oil and grape seed oil, which are valued for both their effectiveness as carriers and their inherent beneficial properties.

Considering the aforementioned factors, this study aimed to perform solvent extraction of compounds from *Cannabis sativa* L. roots using hemp seed oil (HSO), MCT coconut oil (MCT), and grape seed oil (GSO) at different temperatures. Following extraction, the identification and quantification of the extracted compounds were conducted using GC–MS analysis. The concentrations of the target compounds, Friedelin and Carvone, obtained for each solvent and temperature were statistically analyzed by ANOVA to evaluate the influence of solvent type and temperature on their levels.

Furthermore, the antioxidant activity of the cannabis root extracts obtained with the three solvent oils across the different temperatures was determined using the DPPH assay, ABTS test and β-carotene bleaching method. ANOVA was also performed on the antioxidant activity data to assess the impact of solvent and temperature.

Ultimately, this research sought to provide statistically validated evidence regarding the feasibility of extracting bioactive compounds from cannabis roots using edible oils and to confirm the antioxidant properties of these extracts, contributing to the scientific basis for potential cosmetic or medicinal applications.

## 2. Results and Discussion

### 2.1. Particle Size Characterization of Cannabis Roots

Given the cylindrical morphology of *Cannabis sativa* L. roots, their size was described in terms of diameter and length. Prior to grinding, the average diameter and length of the raw roots were determined as 0.36 ± 0.05 mm and 243 ± 22 mm, respectively.

**Figure 1:**
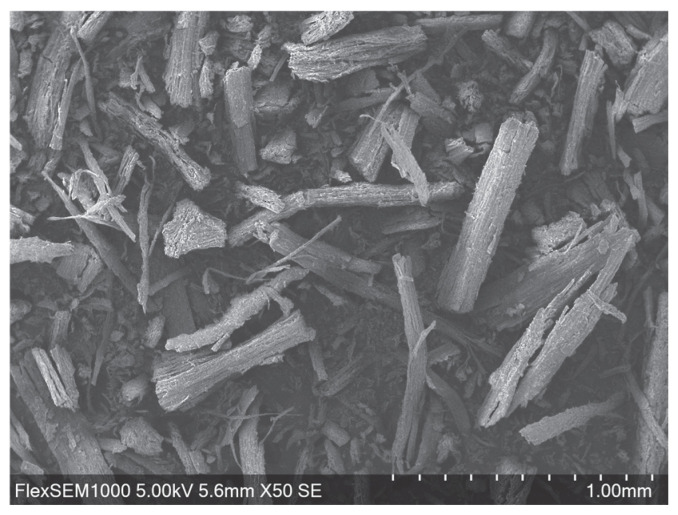
Scanning electron microscopy (SEM) image of finely ground *Cannabis sativa* L. root material, illustrating particle morphology of the fine fraction used for extraction.

**Figure 2:**
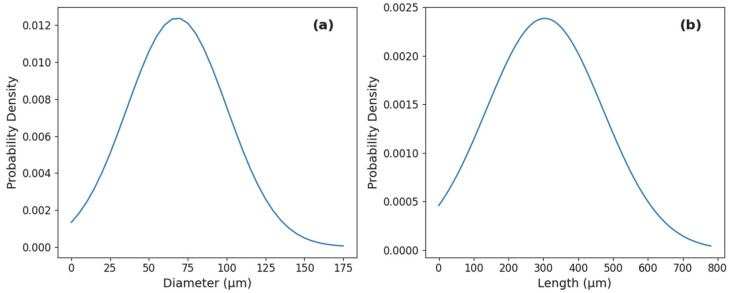
Particle size distribution of ground cannabis roots: (**a**) normal distribution of particle diameter, with an average of 68 ± 32 µm; (**b**) normal distribution of particle length, with an average of 304 ± 167 µm.

Following the grinding process, SEM (Scanning electron microscopy) analysis was employed to evaluate the morphology and dimensions of the resulting fine fraction. The ground root particles maintained a reduced cylindrical shape (Figure 1). Based on the analysis of the representative samples, the particle size followed a normal distribution for both dimensions. The post-grinding diameter was determined to be approximately 68 ± 32 µm (Figure 2a), while the length was measured at approximately 304 ± 167 µm (Figure 2b).

### 2.2. Identified Compounds in Cannabis sativa Extracts 

Gas chromatography–mass spectrometry (GC/MS) analysis of the cannabis root extracts revealed a range of bioactive compounds, primarily fatty acids, aldehydes, and triterpenoids (representative chromatograms are presented in Supplementary Figures S1–S9). A total of 12 compounds were identified across the samples, including the target compound Friedelin and its hydroxylated derivative, Friedelan-3β-ol or Friedelan-3α-ol, which exhibits similar pharmacological properties [22]. Table 1 summarizes the concentrations (mg/g of dry roots) of the detected compounds.

Among the identified constituents, octanoic acid [23,24,25], n-decanoic acid [26,27,28], n-hexadecanoic acid [29,30,31], and heptadecanoic acid [32,33] are saturated fatty acids associated with antimicrobial, antioxidant and anti-inflammatory properties. The extracts also contained several unsaturated fatty acid derivatives such as 9,12-octadecadienoic acid (Z,Z) (linoleic acid) [34], methyl (9Z)-9-octadecenoate (methyl oleate) [35], cis-vaccenic acid [36], and 9,12-octadecadienoic acid (Z,Z)-, methyl ester [37], which are known for their antioxidant and skin-conditioning effects. In addition, the aldehydes 2,4-decadienal [38] and its (E,E)-isomer [39]—volatile products of lipid oxidation—were also identified and have been associated with antimicrobial and cytotoxic potential. Notably, Carvone, another compound initially targeted for extraction, was not detected in any of the extracts under the experimental conditions. Consequently, only Friedelin concentrations were considered for subsequent analysis.

**Figure 3:**
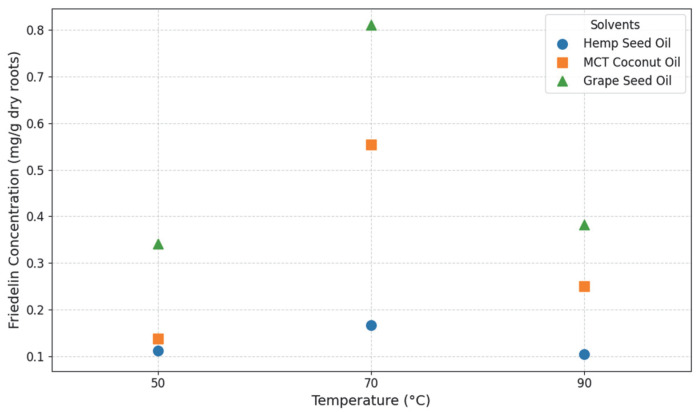
Friedelin concentration (mg/g dry roots) extracted from *C. sativa* roots as a function of temperature (50–90 °C) for the three solvent oils (HSO, MCT, and GSO).

All three solvent oils (Hemp seed oil, MCT coconut oil, and Grape seed oil) successfully extracted Friedelin from the cannabis roots. To visualize the influence of solvent type and temperature on the extraction yield, the Friedelin concentration was plotted against temperature for each oil (Figure 3). As shown in the figure, the optimal extraction temperature for Friedelin was consistently 70 °C across all three solvents. At this temperature, the maximum Friedelin concentrations obtained were 0.167 mg/g dry roots for HSO, 0.554 mg/g dry roots for MCT, and 0.810 mg/g dry roots for GSO. This comparison clearly illustrates that among the tested solvents, GSO exhibited the highest extraction efficiency for Friedelin, followed by MCT, and then HSO.

The maximum friedelin yield achieved using GSO (0.810 mg/g dry roots) is notably superior to previously reported values of 0.100–0.709 mg/g dry weight obtained using ethanol extraction of cannabis roots [12]. This direct comparison demonstrates that edible oils, particularly GSO, can match or even exceed the extraction efficiency of organic solvents. The industrial and environmental relevance of this finding is significant; by using edible oils, the process eliminates the need for energy-intensive solvent removal typically required in ethanol-based extractions. Since the oil serves as both the extraction medium and the final carrier for cosmetic formulations, this approach simplifies the process and supports the potential application of these extracts in cosmetic or medicinal formulations, where the direct use of oil-based systems is advantageous.

To assess the impact of solvent type and extraction temperature on Friedelin concentration, a Two-Way ANOVA without replication was performed (please refer to Supplementary Section D, Table S1). The analysis revealed a statistically significant main effect of solvent type on Friedelin concentration (F=7.42, p=0.045). This indicates that the choice of oil significantly influenced the amount of Friedelin extracted from cannabis roots. Conversely, the main effect of extraction temperature on Friedelin concentration was not statistically significant at the α=0.05 level (F=5.76, p=0.066). However, a noticeable trend towards a temperature effect was observed, suggesting a potential influence that might be more apparent under a more relaxed significance threshold (e.g., α=0.10).

The p=0.066 indicates that while temperature influences the extraction process, it is not the primary determinant of yield compared to the solvent type within the tested range 50–90 °C range. The observed peak at 70 °C followed by a decrease at 90 °C suggests a non-linear relationship, potentially due to a balance between increased solubility and the onset of thermal degradation. Under these experimental conditions, the solvent’s lipophilic profile appears to exert a more dominant role in dictating Friedelin extraction efficiency.

**Figure 4:**
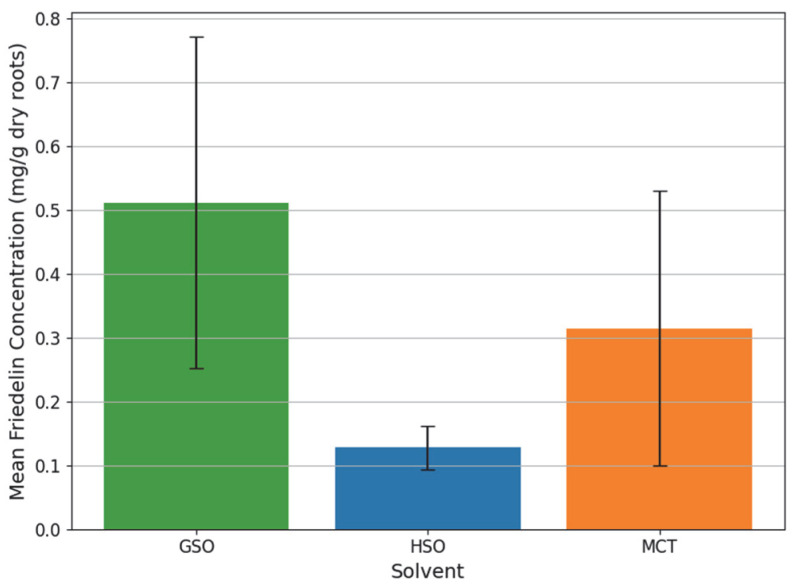
Mean Friedelin concentration (mg/g dry roots) obtained from *Cannabis sativa* L. roots for each solvent oil. Error bars represent the standard deviation across the tested temperatures (50, 70, and 90 °C).

To better visualize the dominant influence of the solvent choice, Figure 4 presents the mean Friedelin concentration for each oil, aggregated across the tested temperatures. GSO demonstrated the highest mean yield, followed by MCT and HSO. This hierarchy is likely dictated by the specific chemical compositions and lipophilic profiles of each oil, which determine their solvation capacity for the non-polar Friedelin.

The superior performance of GSO can be attributed to its unique lipid profile, which is rich in long-chain unsaturated fatty acids, primarily linoleic acid, and phytosterols [40]. These components create a highly lipophilic environment that is exceptionally favorable for the solubilization of non-polar molecules like friedelin. Unlike the other oils tested, GSO also contains a diverse array of phenolic compounds and vitamins that contribute to a unique solvation matrix.

In contrast, MCT coconut oil yielded intermediate Friedelin concentrations. Its composition, dominated by medium-chain triglycerides (C8 caprylic and C10 capric acids), provides a different solvation environment compared to the longer-chain oils [41]. While also non-polar, the shorter hydrocarbon chains of MCTs may present a less optimal affinity or solvency power for the large, complex structure of Friedelin compared to the long chains found in GSO.

Finally, HSO was the least effective solvent for friedelin in this study, despite its high content of essential fatty acids such as linoleic, alpha-linolenic and gamma-linolenic acids [42]. Although HSO shares a similar long-chain profile with GSO, subtle differences in triglyceride arrangements or interactions with other co-extracted compounds may have limited its specific solvency power for friedelin under these experimental conditions.

Regarding selectivity, the GC/MS analysis also revealed differences in the co-extraction of other compounds. While GSO achieved the highest Friedelin yield, it also extracted a broader profile of nine additional compounds, including vaccenic acid, decadienal, and octadecadienoic acid derivatives. In contrast, MCT and HSO demonstrated higher selectivity toward Friedelin, extracting only three and four additional compounds, respectively. Therefore, in terms of selectivity for Friedelin, MCT emerged as the most selective solvent, followed by HSO, with GSO displaying the lowest selectivity. From a practical perspective, this difference in selectivity offers flexibility depending on the intended application. The high selectivity of MCT could be advantageous when the goal is the specific isolation or enrichment of friedelin. Whereas broader co-extraction, as observed with GSO, may be desirable when the objective is to obtain a more complex mixture of bioactive compounds. In this context, the higher solvency of GSO may be beneficial for applications where the combined activity of multiple components is of interest.

### 2.3. Antioxidant Activity of Cannabis Root Extracts

**Figure 5:**
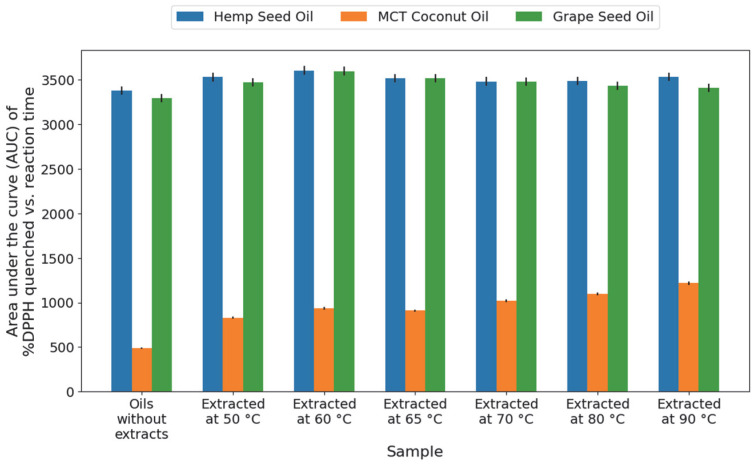
AUC values (area under the curve of % DPPH quenched vs. reaction time) for the pure carrier oils and the oil–extract mixtures obtained at six different extraction temperatures.

**Figure 6:**
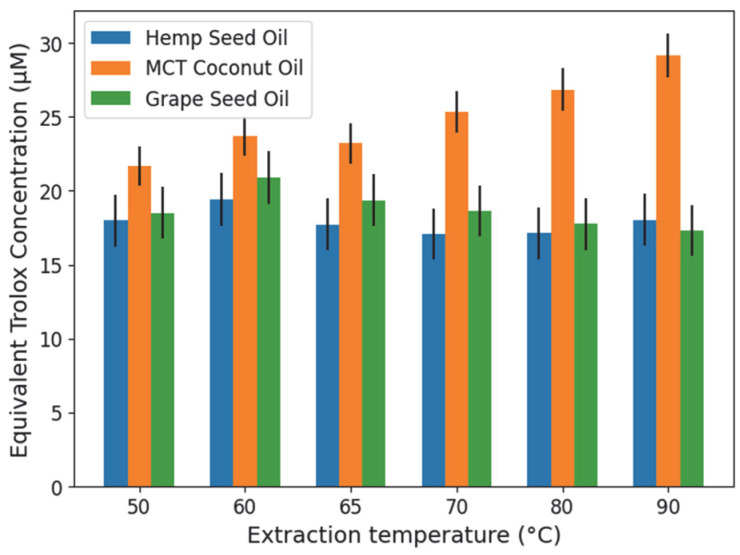
RDSC (Relative DPPH Scavenging Capacity) values of *Cannabis sativa* L. root extracts, expressed as micromoles of Trolox equivalents per liter (µM), obtained at six extraction temperatures using three carrier oils. Uncertainty details calculations provided in Supplementary Section E.

The antioxidant capacity of the cannabis root oil extracts was evaluated using the DPPH assay, ABTS test, and β-carotene bleaching method. For the DPPH assay, Figure 5 presents the AUC (area under the curve of % DPPH quenched vs. reaction time) values obtained for all oil–extract mixtures, together with the intrinsic antioxidant capacity of the oils alone. After accounting for this intrinsic contribution, the RDSC (Relative DPPH Scavenging Capacity) values corresponding to the extracts are shown in Figure 6.

As shown in Figure 5, the combined oil–extract mixtures of HSO and GSO exhibited the highest AUC values, with the maximum activity observed in the HSO sample extracted at 60 °C, followed by GSO at 60 °C. However, the antioxidant activity of HSO and GSO alone was also relatively high, reflecting the inherent antioxidant properties of these carrier oils. In contrast, MCT oil showed low intrinsic antioxidant activity. After correcting for this contribution, the extracts obtained with MCT displayed higher RDSC values (Figure 6), indicating that the presence of the root extract significantly enhanced the antioxidant potential of the MCT system compared to the other oils. Within the MCT group, a peak in antioxidant contribution was observed in the extract obtained at 90 °C.

**Figure 7:**
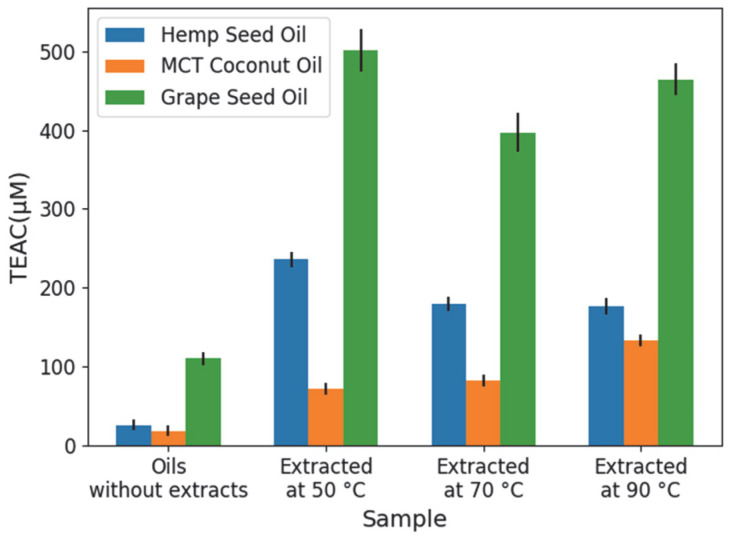
ABTS antioxidant capacity, expressed as TEAC (Trolox Equivalent Antioxidant Capacity) values, for the pure carrier oils and oil–extract mixtures obtained at three extraction temperatures (50, 70, and 90 °C). Details on the standard curve are provided in Supplementary Section B.

For the ABTS test, Figure 7 shows the TEAC (Trolox Equivalent Antioxidant Capacity) values obtained for the oil–extract mixtures and the intrinsic capacity of the carrier oils alone. Regarding this intrinsic activity, GSO exhibited the highest antioxidant capacity (110 µM), followed by HSO (25 µM) and MCT (18 µM). However, a substantial increase in antioxidant activity was observed across all solvents upon the addition of the cannabis root extracts. The most significant enhancement occurred in the GSO extracts, which showed an increase of 280–390 µM compared to the oil alone, reaching the highest value for the extract obtained at 50 °C. The HSO extracts also demonstrated a considerable increase in antioxidant capacity, with values rising by 150 µM–200 µM. In the case of MCT, the extracts enhanced the antioxidant capacity to a lesser extent, with increases ranging from 50 µM to 110 µM. Unlike the DPPH results where MCT extracts showed the highest relative contribution, the ABTS method suggests that the root extracts provide the greatest absolute improvement to the antioxidant profile of the GSO system.

**Figure 8:**
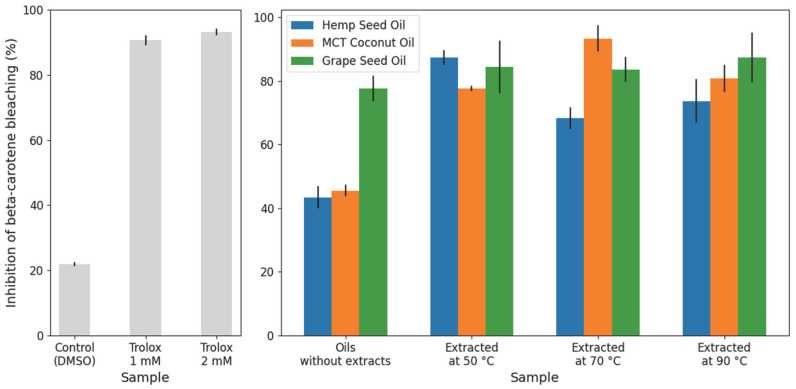
β-carotene bleaching assay results, expressed as percentage of inhibition, for the pure carrier oils and oil–extract mixtures obtained at three extraction temperatures (50, 70, and 90 °C). Trolox and the solvent are included as positive and negative controls, respectively.

For the β-carotene bleaching method, Figure 8 presents the percentage of inhibition obtained for all oil–extract mixtures, the carrier oils alone, and the controls—using Trolox as a positive control and the solvent as a negative control. All samples exhibited a higher inhibition percentage than the negative control, indicating that all tested extracts possess positive antioxidant activity. Regarding the intrinsic antioxidant activity of the oils alone, GSO showed the highest inhibition (77%), followed by MCT (45%) and HSO (43%). Similar to the results in the previous assays, the addition of cannabis root extracts improved the antioxidant capacity of all solvents. However, because GSO already displayed a high baseline activity, the specific enhancement provided by the extracts was less pronounced in this solvent, increasing the inhibition by only 6–10%. In contrast, the HSO and MCT systems showed more substantial improvements, with the extracts increasing inhibition by 25–44% and 32–48%, respectively. The highest overall inhibition percentage was achieved by the MCT extract obtained at 70 °C, followed by HSO at 50 °C and GSO at 90 °C.

A Two-Way ANOVA with replication was conducted to assess the effects of solvent type, extraction temperature, and their interaction on the antioxidant results (RDSC, TEAC, and % Inhibition). The analysis revealed a statistically significant effect of solvent type on all antioxidant responses (RDSC, *p* = 3.94 × 10^−8^; TEAC, *p* = 5.44 × 10^−14^; %Inhibition, *p* = 4.56 × 10^−3^), indicating that the choice of oil solvent significantly influenced the antioxidant capacity of the cannabis root extracts. Regarding extraction temperature, a statistically significant effect was observed only for the ABTS test (*p* = 0.0163), while no significant effect was found for the DPPH and β-carotene bleaching methods. Furthermore, the interaction between solvent type and extraction temperature was statistically significant for the ABTS (*p* = 0.0224) and β-carotene results (*p* = 4.00 × 10^−4^), but not for the DPPH assay.

The highly significant effect of solvent type observed across all antioxidant assays indicates that the choice of extraction and carrier oil is a critical factor in determining the measured antioxidant capacity. This influence is likely attributed to the distinct solvency power of each oil, which dictates the concentration and variety of extracted components. Consequently, the measured antioxidant activity represents a composite contribution from multiple compounds, including Friedelin. However, when comparing these results with the quantified Friedelin concentrations, a direct linear correlation was not consistently observed across all samples, despite Friedelin having previously demonstrated positive antioxidant effects in other studies [8].

It is also important to consider that the high intrinsic antioxidant capacity of the pure carrier oils, specifically GSO and HSO, is consistent with literature reports regarding their bioactive properties [40,42]. However, in certain assays like the DPPH test, this elevated background activity may lead to the saturation of the DPPH radical, meaning the system has already reached its maximum scavenging potential. This effect can potentially mask the additional contributions of the extract or limit the measurable increase in activity, making it challenging to quantify the full extent of additive effects within the final oil-based formulation.

Despite these quantification challenges, the overall findings confirm that all cannabis root extracts, whether evaluated as isolated components or within their respective oil vehicles, consistently exhibited positive antioxidant results across the DPPH, ABTS and b-carotene tests. This confirms the widespread presence of compounds with antioxidant capacity in cannabis roots, regardless of the specific oil solvent used for the extraction process.

Beyond antioxidant potential, the identified presence of Friedelin and other compounds in these cannabis root extracts suggests broader therapeutic applications. Friedelin, in particular, has been widely attributed with notable antimicrobial and anti-inflammatory properties. Given that various other compounds identified in these extracts also possess documented bioactivities, future research should focus on comprehensively assessing the antimicrobial and anti-inflammatory activities of these oil extracts. Demonstrating such properties would further support the potential use of *Cannabis sativa* L. root extracts in broader medicinal or therapeutic applications.

## 3. Materials and Methods

### 3.1. Plant Material-Cannabis Roots

**Figure 9:**
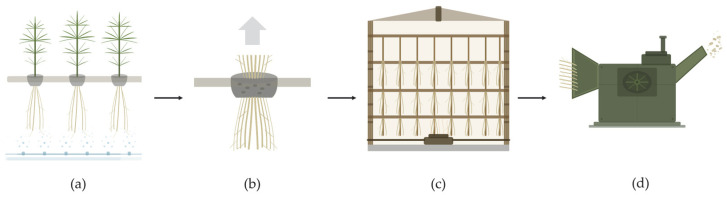
Processing stages of *Cannabis sativa* L. roots. Arrows indicate the sequential flow of operations: (**a**) aeroponic cultivation of hemp plants; (**b**) plant harvest; (**c**) drying in a tobacco kiln; and (**d**) mechanical chipping using an industrial wood chipper.

Industrial hemp (*Cannabis sativa* L.) plants containing less tan 0.3% THC (*w*/*w*) were aeroponically grown at a farm in St. Thomas, Ontario, Canada. The cultivation was conducted in accordance with the Canadian Cannabis Act and the Industrial Hemp Regulations (SOR/2018-145) under a valid Health Canada industrial hemp license [43]. The plants were harvested in September 2022, dried in a tobacco kiln, and subsequently chipped using an industrial wood chipper (Figure 9). The chipped roots were stored at room temperature until use, with a measured moisture content of 5.5%.

For the extraction process, the dried chipped roots were ground using a Dade DF-15 impact grinder (Wenling Linda Machinery Co., Ltd., Wenling City, China), and only the fine fraction obtained was used. The average particle size of the ground material was determined through morphological analysis using a scanning electron microscope (SEM, Hitachi FlexSEM 1000; Hitachi High-Tech Corporation, Minato-ku, Tokyo, Japan) operating at 5 kV. SEM images of a representative sample were analyzed using ImageJ software (version 1.54g) to measure particle dimensions.

### 3.2. Chemicals

Refined hemp seed oil (Netherlands), MCT 60/40 fractionated coconut oil (Malaysia), and refined cosmetic-grade grape seed oil (Chile) were obtained from New Directions Aromatics Inc. (Mississauga, ON, Canada). Ethanol (95%) was provided by Commercial Alcohols (Brampton, ON, Canada). Chloroform (>99.8%) and ethyl acetate (≥99.5%) were purchased from Fisher Chemical (Fair Lawn, NJ, USA). Dichloromethane (≥99.5%), dimethyl sulfoxide (DMSO, ≥99%), mineral oil, linoleic acid (≥95.0%), and Tween 40 (polyoxyethylenesorbitan monopalmitate, ~90.0%) were obtained from Sigma-Aldrich (St. Louis, MO, USA).

Friedelin (analytical standard, ≥95.0%), Trolox ((±)-6-hydroxy-2,5,7,8-tetramethylchromane-2-carboxylic acid, 97%), DPPH (2,2-diphenyl-1-picrylhydrazyl, Quality Level 100), ABTS (2,2′-azino-bis(3-ethylbenzothiazoline-6-sulfonic acid) diammonium salt, ≥98%), potassium persulfate (≥99.0%), and β-carotene (≥93%) were also purchased from Sigma-Aldrich (St. Louis, MO, USA). Oxygen gas (O_2_, 99.6%) was supplied by Linde Canada Inc. (Mississauga, ON, Canada).

### 3.3. Extraction

Hemp seed oil, MCT coconut oil, and grape seed oil were selected as the extraction solvents. Extractions were performed at six different temperatures: 50, 60, 65, 70, 80, and 90 °C. Each extraction used 5 g of ground cannabis roots and approximately 40 mL of solvent oil, corresponding to a 2:1 solvent-to-root volume ratio. This ratio was selected based on preliminary observations of solvent absorption by the root matrix to ensure complete immersion of the solid material throughout the extraction process.

The roots and solvent were initially blended using a vortex mixer for 1 min. The extraction was performed using a heating and stirring plate equipped with a thermocouple to monitor the temperature of a mineral oil bath (bain-marie). The desired extraction temperature was set on the heating plate. Once the temperature of the oil bath stabilized, an Erlenmeyer flask containing the root-solvent mixture was immersed in the oil bath and stirred continuously using a magnetic stir bar for 24 h. Following the 24 h period, the heating was discontinued, and the Erlenmeyer flask was removed from the bath. The mixture was then filtered using a Büchner funnel and Whatman No. 1 filter paper (pore size: 11 µm). The filtered oil containing the extracted compounds was collected for subsequent analysis.

### 3.4. Gas Chromatography-Mass Spectrometry (GC-MS) Analysis

The extracted compounds were identified and quantified by analyzing both the extracted samples and the pure solvent oils with Gas Chromatography-Mass Spectrometry (GC-MS). For this, the extracted oil samples were dissolved in dichloromethane before being injected into an Agilent GC/MS (7890A GC and 5975C MSD; Agilent Technologies, Inc., Santa Clara, CA, USA) system. After this, 1 µL of sample solution was injected by an autosampler in splitless mode to an Agilent J&W HP-5 ms 19091S-433 GC column (30 m × 250 µm × 0.25 µm). Helium was used as the carrier gas at a constant flow rate of 1.4 mL/min. The injector temperature was maintained at 250 °C while the oven temperature was programmed with an initial temperature of 40 °C for 1 min, then ramped at 7 °C/min to 250 °C, and further at 10 °C/min to 310 °C, where it was held for 23 min.

The MS detector (MSD) worked with electron ionization where the m/z scan range was 33–600 amu after a solvent delay of 3.2 min. The temperatures of the MSD transfer line, MS source, and MS quad were 280, 230, and 150 °C, respectively. Moreover, a standard sample of friedelin was employed for calibration and quantification while other components were identified using the NIST 23 MS library and semi-quantified based on the response of the friedelin standard.

Quantification results were initially calculated as concentrations within the oil extract (ppm, mg/kg) and subsequently converted to milligrams of compound per gram of dry root material (mg/g dry roots) for final reporting and comparison of extraction efficiency.

### 3.5. Antioxidant Tests

The antioxidant capacity of the edible oil-based cannabis root extracts was evaluated using three complementary assays. Because antioxidant activity can occur through various chemical pathways, such as radical scavenging and the inhibition of lipid peroxidation, no single method can fully characterize the antioxidant profile of a complex plant extract. To ensure a comprehensive assessment, both SET-based methods (DPPH and ABTS) and a HAT-based method (β-carotene bleaching) were employed. This multi-method approach allows for the evaluation of the extracts’ ability to neutralize free radicals in different environments and their effectiveness in protecting lipid systems from oxidative degradation.

#### 3.5.1. DPPH Assay

The antioxidant capacity of the cannabis root extracts was first evaluated using the DPPH assay, with Trolox used as the antioxidant standard. A 0.2 mM DPPH working solution was prepared by dissolving 1.183 mg of DPPH in 15 mL of ethyl acetate. For the calibration curve, a 0.5 mM Trolox stock solution was prepared by dissolving 1.877 mg in 15 mL of ethyl acetate, with serial dilutions performed to obtain working solutions in the 7–500 µM range. All absorbance measurements were recorded at 515 nm using a UV-Vis NIR spectrophotometer (UV-3600; Shimadzu Corporation, Kyoto, Japan) to monitor the reduction of the DPPH radical [17,19].

The assay began by establishing a blank measurement with 2000 µL of ethyl acetate. For each sample reaction mixture, 500 µL of the root extract solution was mixed with 500 µL of ethyl acetate. Subsequently, 1000 µL of the 0.2 mM DPPH solution was added, and the mixture was vortexed for 5 s. The reaction mixture was immediately transferred to a cuvette, and absorbance was measured every minute for 40 min. This procedure was consistently applied to all 18 cannabis root extract samples.

Negative control absorbances were determined by substituting the root extract with 500 µL of ethyl acetate in the first step of the procedure. Positive standard control absorbances were determined similarly, using the prepared Trolox solutions instead of the root extracts. To ensure reliability, all assays were conducted in triplicate.

The percentage of DPPH quenched at each time point was calculated for all standards and samples using the following equation:

(1) $${\%\;DPPH\;quenched}_{i}=\left(1-\frac{A_{sample,i}-A_{blank}}{A_{control,i}-A_{blank}}\right)\times 100$$

where $A_{sample,i}$ is the absorbance of sample at reaction time i, $A_{blank}$ is the absorbance of the blank, and $A_{control,i}$ is the absorbance of the negative control at reaction time i.

To integrate both the kinetic and thermodynamic behavior of the antioxidant-DPPH reaction, the area under the curve (AUC) of % DPPH quenched versus reaction time was calculated for each standard concentration and sample as follows:

(2) $$AUC={0.5(\%\;DPPH\;quenched}_{0})+\sum_{i=1}^{39}{\%\;DPPH\;quenched}_{i}+{0.5(\%\;DPPH\;quenched}_{40})$$

The % DPPH quenched and AUC values were calculated in the same manner for all pure solvent oil samples (without extracts) to determine their inherent antioxidant contribution.

A standard curve was constructed by plotting the AUC values from the Trolox standards against their corresponding concentrations. From the linear portion of this curve, a linear regression equation was derived, which allowed calculation of the Relative DPPH Scavenging Capacity (RDSC) of the tested samples with respect to Trolox.

To account for the intrinsic antioxidant capacity of the solvent oils, the net AUC of the extracts was determined by subtracting the AUC of the pure oil from the AUC of the corresponding oil–extract mixture:

(3) $${AUC}_{extracts}={AUC}_{oil\;with\;extracts}-{AUC}_{oil\;without\;extracts}$$

Using these net AUC values for the extracts and the linear regression equation from the Trolox standard curve, the equivalent Trolox concentration (micromoles Trolox/Liter) was calculated for each extract sample. This value represents the Relative DPPH Scavenging Capacity (RDSC) of the extract with respect to Trolox.

#### 3.5.2. ABTS Test

The ABTS radical cation (${ABTS}^{•+}$) was produced by reacting 7 mM ABTS with 2.45 mM potassium persulfate (final concentration) in water. This stock solution was kept at room temperature in the dark for 16 h. The working solution was then prepared by diluting the stock solution with ethanol to an absorbance of 0.700 ± 0.02 at 734 nm [18,44]. For the calibration curve, a 5 mM Trolox stock solution was prepared by dissolving 25.7 mg in 20 mL of ethyl acetate, with serial dilutions performed to obtain working solutions in the 20–600 µM range.

For each sample, 500 µL of the root extract oil was mixed with 1000 µL of ethyl acetate. Subsequently, 70 µL of this extract sample solution was added to 1330 µL of the ABTS working solution. The reaction mixture was gently mixed, incubated in the dark at room temperature for 30 min, and the absorbance was then measured at 734 nm using the same UV–Vis NIR spectrophotometer described above [18,44,45]. This procedure was applied to all cannabis root extract samples, as well as to the three solvent oils without extracts, to determine their inherent antioxidant contribution.

Negative control absorbance was determined by substituting the extract sample solution with 70 µL of ethyl acetate. Positive control absorbances were determined similarly, using the prepared Trolox solutions instead of the extract sample solutions. To ensure reliability, all assays were conducted in triplicate.

The percentage of Inhibition was calculated for all Trolox standard solutions and sample solutions (root extracts and pure solvent oils) using the following equation:

(4) $$\%\;Inhibition=\left(\frac{A_{control}-A_{sample}}{A_{control}}\right)\times 100$$

where $A_{control}$ is the absorbance of the negative control and $A_{sample}$ is the absorbance of the testing sample.

A standard curve was constructed by plotting the % Inhibition values of the Trolox standards against their corresponding concentrations. A linear regression equation was obtained and used to calculate the TEAC (Trolox Equivalent Antioxidant Capacity) of all root extracts and pure solvent oils.

#### 3.5.3. β-Carotene Bleaching Method

The antioxidant activity was further evaluated by the β-carotene bleaching method, which measures the ability of the samples to prevent the bleaching of β-carotene in a lipid peroxidation system.

A β-carotene solution was first prepared by dissolving 10 mg of β-carotene in 10 mL of chloroform. Then, 25 µL of linoleic acid and 200 mg of Tween 40 were added. The chloroform was completely removed using a rotary evaporator under reduced pressure. In parallel, 100 mL of distilled water was saturated with oxygen for 30 min at a flow rate of 100 mL/min. This oxygen-saturated water was then slowly added to the β-carotene residue with vigorous agitation to form a stable working emulsion, which was used immediately [16,45,46].

Trolox was used as a positive control. A 10 mM stock solution was prepared in DMSO and further diluted to obtain 1 mM and 2 mM working solutions.

For each sample, 500 µL of the root extract oil was mixed with 1000 µL of DMSO. Subsequently, 350 µL of this extract sample solution was added to 2.5 mL of the β-carotene emulsion. The reaction mixture was incubated in a hot water bath at 45 °C for 3 h, with absorbance measured at 470 nm at the beginning (t = 0) and after 3 h (t = 180 min) using the same UV–Vis NIR spectrophotometer described above [16,46]. This procedure was applied to all cannabis root extract samples, as well as to the three solvent oils without extracts, to determine their inherent antioxidant contribution.

Negative control absorbance was determined by substituting the extract sample solution with 350 µL of DMSO. Positive control absorbances were determined similarly, using the prepared Trolox solutions instead of the extract sample solutions. A zeroing emulsion (blank) was prepared following the same procedure but omitting the β-carotene to account for any background interference. To ensure reliability, all assays were conducted in triplicate.

The antioxidant activity, expressed as the percentage of inhibition, was calculated for all Trolox standard solutions and sample solutions (root extracts and pure solvent oils) using the following equation:

(5) $$\%\;Inhibition=\left(\frac{A_{t}}{A_{0}}\right)\times 100$$

where $A_{t}$ is the absorbance of β-carotene remaining in the sample after 3 h and $A_{0}$ is the absorbance of β-carotene at the beginning of the test [46].

### 3.6. Statistical Analysis

Analysis of Variance (ANOVA) was employed to statistically evaluate the effects of extraction solvent and temperature on both Friedelin concentration and the antioxidant activity of *Cannabis sativa* L. root extracts. The independent factors investigated were solvent type (hemp seed oil, MCT coconut oil, and grape seed oil) and extraction temperature (50, 70, and 90 °C). The dependent variables were Friedelin concentration and antioxidant capacity (expressed as Trolox equivalents and %Inhibition).

For the Friedelin concentration data, a Two-Way ANOVA without replication was conducted, as each unique solvent-temperature combination represented a single measurement. For the antioxidant activity data, a Two-Way ANOVA with replication was performed, since antioxidant assays were conducted in triplicate. This allowed for the evaluation of the main effects of solvent and temperature, as well as their interaction effect. Following the ANOVA, if a significant main effect was identified, Post hoc Tukey’s HSD tests were conducted to determine specific pairwise differences between group means.

Prior to conducting the ANOVA, the assumptions of normality of residuals, homogeneity of variances, and independence of residuals were verified for all datasets (details of these checks are provided in Supplementary Section C). Normality was assessed using Q-Q plots and the Shapiro–Wilk test, while homogeneity of variances was evaluated using Levene’s test and visual inspection of residuals and boxplots. Visual inspection of residual plots was also used to confirm the independence of residuals. All assumptions were met, and all statistical analyses were performed using Python (version 3.13) with the statsmodels package (scripts for these analyses are available in Supplementary Section D), with a significance threshold set at p<0.05.

## 4. Conclusions

This study successfully investigated the solvent extraction of bioactive compounds from *Cannabis sativa* L. roots using hemp seed oil (HSO), MCT coconut oil (MCT), and grape seed oil (GSO) across various temperatures, aiming to provide statistically validated evidence for their potential application in cosmetic or medicinal formulations.

Regarding the extraction of targeted compounds, all three solvent oils effectively extracted Friedelin from *Cannabis sativa* L. roots. Analysis of Variance (ANOVA) confirmed that solvent type significantly influenced Friedelin concentration, with GSO yielding the highest amounts, followed by MCT and HSO. While the optimal extraction temperature for Friedelin was consistently found to be 70 °C for all solvents, temperature itself did not show a statistically significant main effect on concentration. It is also important to note that Carvone, another compound initially targeted for extraction, was not detected in any of the extracts under the experimental conditions.

Concerning antioxidant properties, all cannabis root extracts, regardless of solvent or temperature, consistently exhibited positive antioxidant activity across the three methods tested: DPPH, ABTS and β-carotene. ANOVA results clearly indicated a statistically significant effect of solvent type on antioxidant activity. GSO, both as a pure oil and in combination with cannabis root extracts, consistently showed higher antioxidant values across the three methods. When comparing Friedelin concentrations with antioxidant results, no direct linear correlation was observed. This suggests that Friedelin is not the sole contributor to the overall antioxidant capacity of the extracts. Instead, the measured values reflect the combined effect of multiple co-extracted compounds. Despite the inherent antioxidant properties of the carrier oils and the potential for assay saturation in combined formulations, the findings confirm that cannabis root extracts possess robust radical scavenging activity and the ability to inhibit lipid peroxidation.

In conclusion, this research validates the feasibility of extracting bioactive compounds from cannabis roots using accessible edible oils, achieving Friedelin recoveries higher than those previously reported for alcoholic extractions. The choice of solvent proved to be a critical factor, affecting both the yield of specific compounds such as Friedelin and the overall antioxidant profile due to the nature of the co-extracted constituents. These findings provide an important foundation for the development of *Cannabis sativa* L. root-based products for cosmetic or medicinal applications.

## Figures and Tables

**Table 1:** Concentrations (mg/g of dry roots) of identified compounds in *Cannabis sativa* L. root extracts obtained using hemp seed oil (HSO), MCT coconut oil (MCT), and grape seed oil (GSO) at 50, 70, and 90 °C.

RT (min)	CAS Number	Compound Name	Concentration (mg/g Dry Roots)								
			HSO 50 °C	HSO 70 °C	HSO 90 °C	MCT 50 °C	MCT 70 °C	MCT 90 °C	GSO 50 °C	GSO 70 °C	GSO 90 °C
11.8	000124-07-2	Octanoic Acid				2.142	18.059	6.171			
13.8	002363-88-4	2,4-Decadienal							0.985	3.856	1.105
14.3	025152-84-5	2,4-Decadienal, (E,E)-							1.355	6.012	1.753
15.4	000334-48-5	n-Decanoic acid				1.188	8.931	3.241			
24.7	000057-10-3	n-Hexadecanoic acid	0.599	0.572	0.367				0.183	0.211	0.118
26.2	000506-12-7	Heptadecanoic acid							0.403	0.158	0.364
26.5	000112-63-0	9,12-Octadecadienoic acid (Z,Z)-, methyl ester							0.296	0.118	0.298
26.6	000112-62-9	Methyl (9Z)-9-Octadecenoate							0.071	0.108	0.304
27	000060-33-3	9,12-Octadecadienoic acid (Z,Z)-	12.630	12.979	8.972				2.045	0.955	0.996
27.1	000506-17-2	cis-Vaccenic acid	4.593	3.966	2.396				4.538	2.302	3.810
40	016844-71-6	Friedelan-3β-ol or Friedelan-3α-ol	0.087	0.137	0.092	0.071	0.701	0.113	0.272	0.580	0.291
40.2	000559-74-0	Friedelin	0.112	0.167	0.104	0.138	0.554	0.250	0.341	0.810	0.382

## Data Availability

Data will be made available on request.

## Supplementary Materials

The following supporting information can be downloaded at: https://www.mdpi.com/article/10.3390/molecules31091473/s1, Figure S1: GC/MS chromatogram of the extracted sample using HSO at 50 °C; Figure S2: GC/MS chromatogram of the extracted sample using HSO at 70 °C; Figure S3: GC/MS chromatogram of the extracted sample using HSO at 90 °C; Figure S4: GC/MS chromatogram of the extracted sample using MCT at 50 °C; Figure S5: GC/MS chromatogram of the extracted sample using MCT at 70 °C; Figure S6: GC/MS chromatogram of the extracted sample using MCT at 90 °C; Figure S7: GC/MS chromatogram of the extracted sample using GSO at 50 °C; Figure S8: GC/MS chromatogram of the extracted sample using GSO at 70 °C; Figure S9: GC/MS chromatogram of the extracted sample using GSO at 90 °C; Figure S10: Dynamic assessment of DPPH radical scavenging activity: A representative example from *Cannabis sativa* L. root extract obtained with MCT at 90 °C; Figure S11: Trolox standard curve for the Relative DPPH Scavenging Capacity (RDSC) method; Figure S12: Trolox standard calibration curve for the ABTS radical cation scavenging assay.; Figure S13: Python code for Shapiro–Wilk test and Q-Q plot generation for Friedelin concentration residuals; Figure S14: Q-Q plot of residuals for Friedelin concentration data; Figure S15: Python code for generating the residuals vs. fitted values plot for Friedelin concentration data; Figure S16: Residuals vs. fitted values plot for Friedelin concentration data; Figure S17: Python code for Shapiro–Wilk test and Q-Q plot generation for antioxidant activity data residuals; Figure S18: Q-Q plot of residuals for RDSC values; Figure S19: Q-Q plot of residuals for TEAC values.; Figure S20: Q-Q plot of residuals for % Inhibition values.; Figure S21: Python code for Levene’s test and boxplot generation for antioxidant activity data; Figure S22: Boxplot showing variances of RDSC data across groups; Figure S23: Python code for plotting residuals vs. fitted values for antioxidant activity data; Figure S24: Residuals vs. fitted values plot for RDSC data.; Figure S25: Residuals vs. fitted values plot for TEAC data.; Figure S26: Residuals vs. fitted values plot for % Inhibition data.; Figure S27: Python code for Two-Way ANOVA without replication for Friedelin concentration data; Figure S28: Python code for Two-Way ANOVA with replication for antioxidant activity data; Figure S29: Python code for Post hoc Tukey’s HSD tests for the effect of solvent type on RDSC.; Figure S30: Tukey HSD Simultaneous Confidence Interval Plot for RDSC values; Table S1: Summary of Two-Way ANOVA without replication results, detailing the F-values, degrees of freedom, and *p*-values for the effects of solvent type and extraction temperature on Friedelin concentration (ppm) in *Cannabis sativa* L. root extracts.; Table S2: Summary of Two-Way ANOVA with replication results, detailing the F-values, degrees of freedom, and *p*-values for the individual and interactive effects of solvent type and extraction temperature on Relative DPPH Scavenging Capacity (RDSC) (µmol Trolox equivalent per liter) in *Cannabis sativa* L. root extracts.; Table S3: Summary table presenting the results of the Post hoc Tukey’s HSD tests for solvent type on RDSC values.; Table S4: Summary of Two-Way ANOVA with replication results, detailing the F-values, degrees of freedom, and *p*-values for the individual and interactive effects of solvent type and extraction temperature on TEAC values, for the ABTS test.; Table S5: Summary of Two-Way ANOVA with replication results, detailing the F-values, degrees of freedom, and *p*-values for the individual and interactive effects of solvent type and extraction temperature on % Inhibition values, for β-carotene bleaching method.

## Abbreviations

The following abbreviations are used in this manuscript:

HSO	Hemp seed oil
MCT	MCT coconut oil
GSO	Grape seed oil
SFE-CO_2_	Supercritical CO_2_ extraction
DMSO	Dimethyl sulfoxide
ANOVA	Analysis of Variance
SET	Single Electron Transfer
HAT	Hydrogen Atom Transfer
DPPH	2,2-diphenyl-1-picrylhydrazyl
ABTS	2,2′-azino-bis(3-ethylbenzothiazoline-6-sulfonic acid)
FRAP	Ferric reducing antioxidant power
AUC	Area Under the Curve of % DPPH quenched vs. reaction time
RDSC	Relative DPPH Scavenging Capacity
TEAC	Trolox Equivalent Antioxidant Capacity
GC-MS	Gas Chromatography-Mass Spectrometry
MSD	MS detector
SEM	Scanning Electron Microscope
