# Supplementary material for: Edible Oil-Based Extraction of Cannabis sativa L. Roots: Effect of Solvent and Temperature on Friedelin Yield and Antioxidant Activity
Source: Molecules. 2026 Apr 29;31(9):1473. doi: 10.3390/molecules31091473 (PMC13165115; doi:10.3390/molecules31091473)
Supplement: Supplementary file 1 [file molecules-31-01473-s001.zip › Supplementary material.pdf]

Supplementary information

A. GC/MS chromatograms

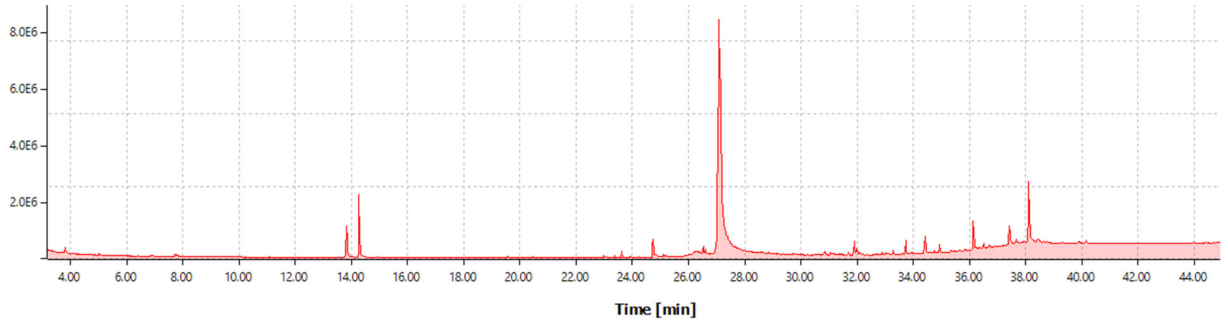

Figure S1. GC/MS chromatogram of the extracted sample using HSO at 50°C.

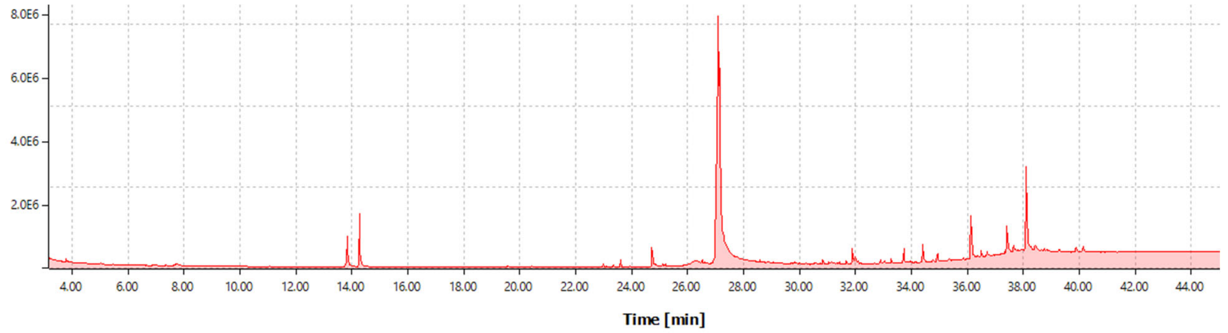

Figure S2. GC/MS chromatogram of the extracted sample using HSO at 70°C.

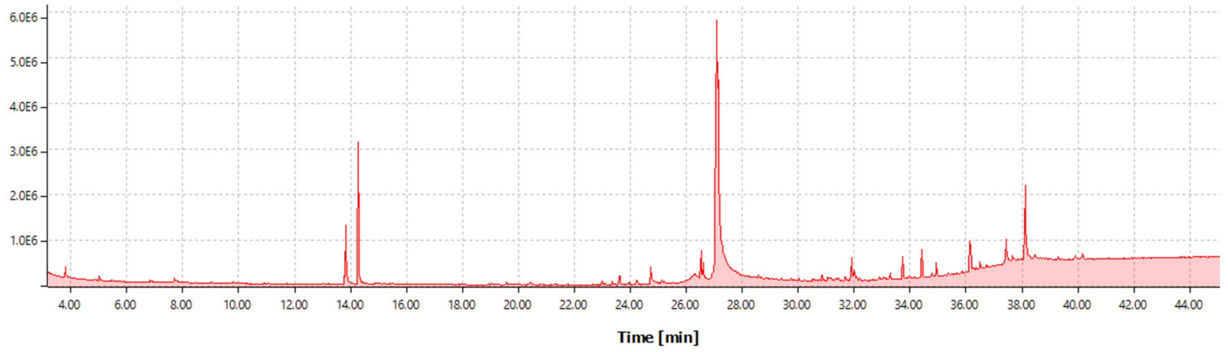

Figure S3. GC/MS chromatogram of the extracted sample using HSO at 90°C.

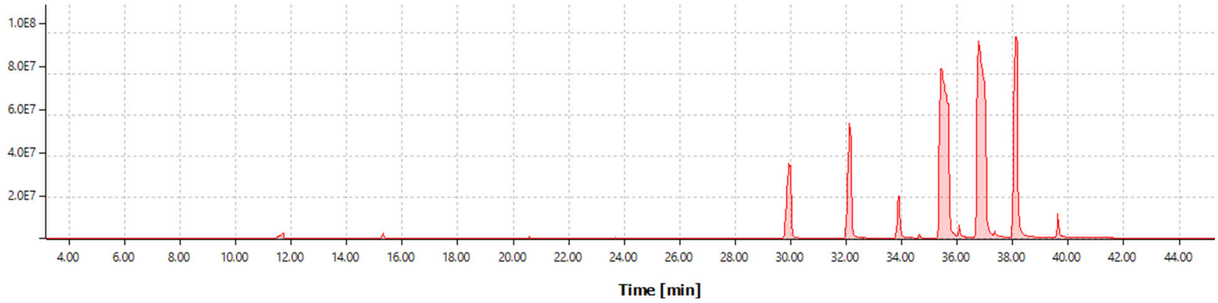

Figure S4. GC/MS chromatogram of the extracted sample using MCT at 50°C.

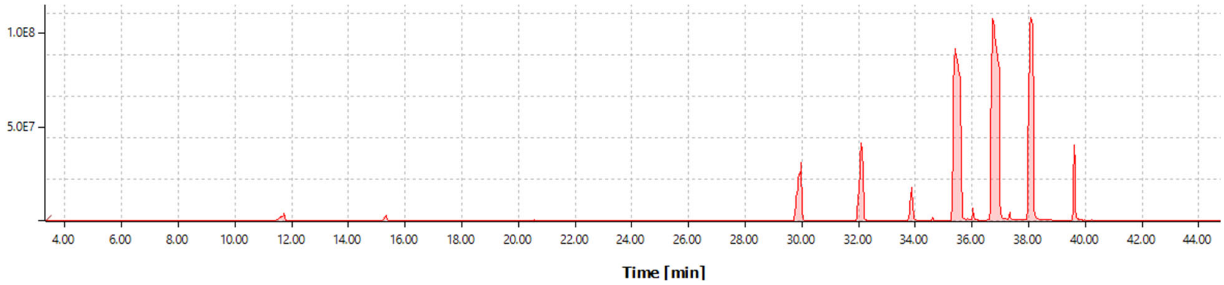

Figure S5. GC/MS chromatogram of the extracted sample using MCT at 70°C.

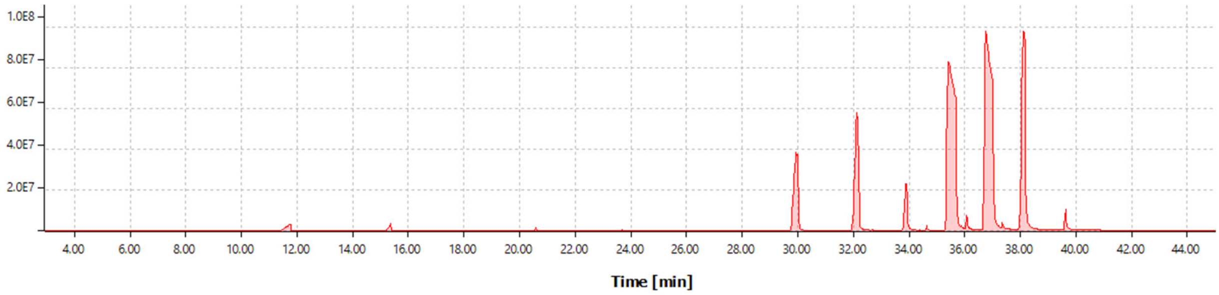

Figure S6. GC/MS chromatogram of the extracted sample using MCT at 90°C.

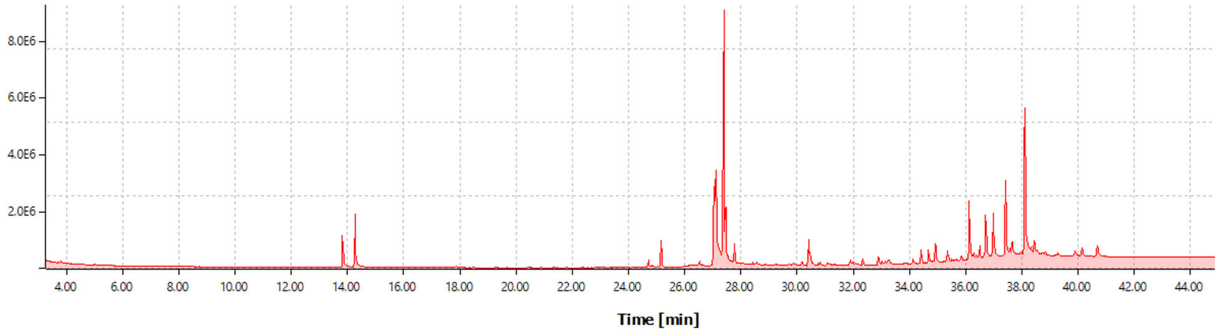

Figure S7. GC/MS chromatogram of the extracted sample using GSO at 50°C.

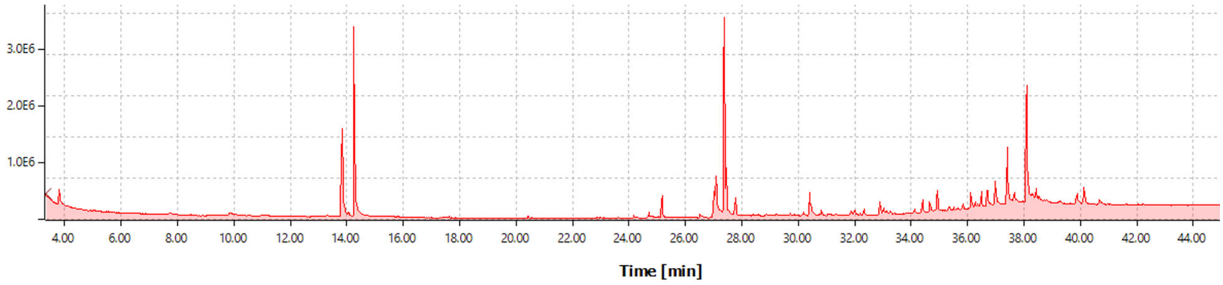

Figure S8. GC/MS chromatogram of the extracted sample using GSO at 70°C

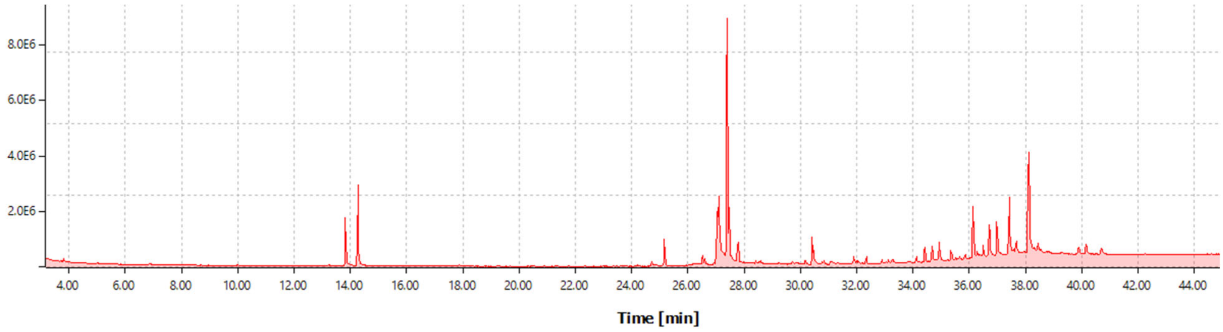

Figure S9. GC/MS chromatogram of the extracted sample using GSO at 90°C

B. Extended Data: Antioxidant Capacity Assays

1. Antioxidant Profiles - DPPH test

To illustrate the kinetic profile of the DPPH assay, Figure S10**Error! Reference source not found.**a presents the absorbance measurements over 40 minutes for a representative sample (MCT extract at 90°C), with Figure S10**Error! Reference source not found.**b showing its corresponding percentage of DPPH quenched over time.

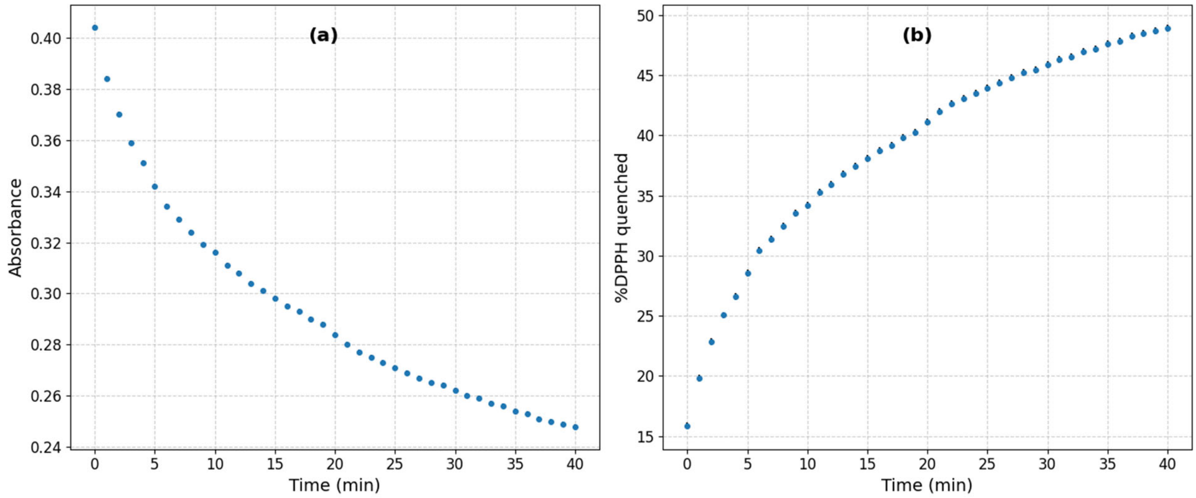

Figure S10. Dynamic assessment of DPPH radical scavenging activity: A representative example from Cannabis sativa L. root extract obtained with MCT at 90°C. ***a)*** *Absorbance values (at 515 nm) recorded over 40 minutes, illustrating the reduction of the DPPH radical.* ***b)*** *Calculated percentage of DPPH quenched over the reaction time, reflecting the extract's antioxidant capacity.*

2. Standard curve - DPPH test

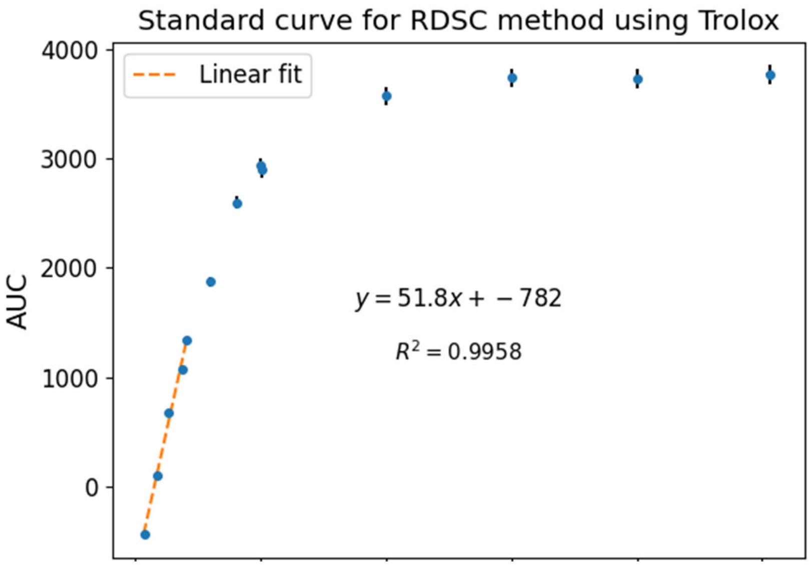

Figure S11. Trolox standard curve for the Relative DPPH Scavenging Capacity (RDSC) method.

Trolox standard curve for the Relative DPPH Scavenging Capacity (RDSC) method was constructed by plotting the AUC values (Area Under the Curve of % DPPH quenched vs. time) against their corresponding Trolox concentrations (µM). A linear regression equation $y = 51.8x - 782$ with a coefficient of determination (R^2) of 0.9958 was derived from the linear portion of this standard curve to enable the quantification of sample antioxidant capacity in Trolox equivalents.

3. Standard curve - ABTS test

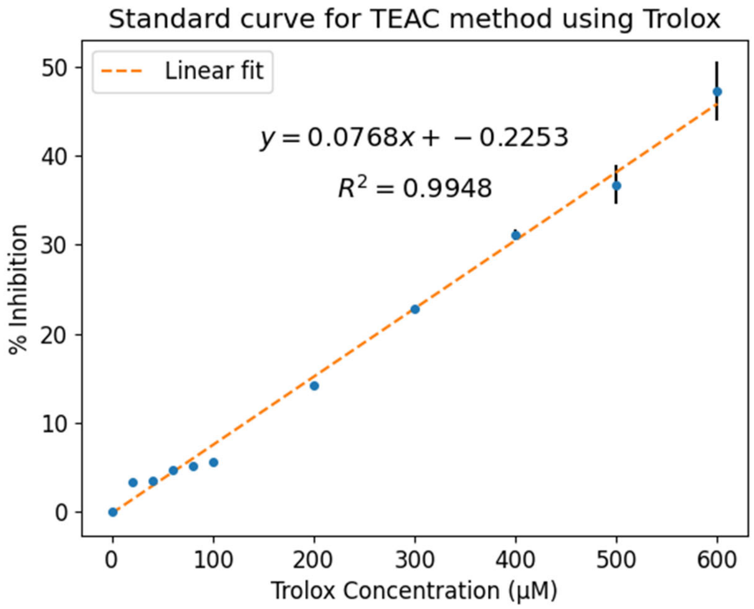

Figure S12. Trolox standard calibration curve for the ABTS radical cation scavenging assay.

Trolox standard calibration curve for the ABTS radical cation scavenging assay was constructed by plotting the percentage of inhibition against Trolox concentration (µM). The resulting linear regression was used to calculate the Trolox Equivalent Antioxidant Capacity (TEAC) of the extracts.

C. Assumptions for ANOVA

Prior to conducting the Analysis of Variance (ANOVA), the underlying assumptions of normality of residuals, homogeneity of variances, and independence of residuals were rigorously verified for all datasets: Friedelin concentration and antioxidant results data (RDSC values, TEAC values, and % of Inhibition).

1. Friedelin Concentration Data

1.1. Normality of residuals:

Normality of residuals was assessed using the Shapiro-Wilk test and a Quantile-Quantile (Q-Q) plot.

```
import pandas as pd
import numpy as np
import scipy.stats as stats
import matplotlib.pyplot as plt

# 1. Organize data

data = {
    'Solvent': ['HSO', 'HSO', 'HSO', 'MCT', 'MCT', 'MCT', 'GSO', 'GSO', 'GSO'],
    'Temperature': [50, 70, 90]*3,
    'Friedelin': [0.112,0.167,0.104,0.138,0.554,0.250,0.341,0.810,0.382]
}
df = pd.DataFrame(data)

# 2. Compute means
grand_mean = df['Friedelin'].mean()
solvent_means = df.groupby('Solvent')['Friedelin'].mean()
temp_means = df.groupby('Temperature')['Friedelin'].mean()

# 3. Predicted values and residuals
def get_predicted(row):
    return solvent_means[row['Solvent']] + temp_means[row['Temperature']] - grand_mean

df['Predicted'] = df.apply(get_predicted, axis=1)
df['Residual'] = df['Friedelin'] - df['Predicted']

# 4. Shapiro-Wilk test for normality of residuals
shapiro_stat, shapiro_p = stats.shapiro(df['Residual'])
print("\nShapiro-Wilk Test:")
print(f"Statistic = {shapiro_stat:.4f}, p-value = {shapiro_p:.4f}")

# 5. QQ Plot
plt.figure()
stats.probplot(df['Residual'], dist="norm", plot=plt)
plt.title("QQ Plot of Residuals")
```

Figure S13. Python code for Shapiro-Wilk test and Q-Q plot generation for Friedelin concentration residuals.

- Shapiro-Wilk Test results: Statistic = 0.9316, p-value = 0.5114

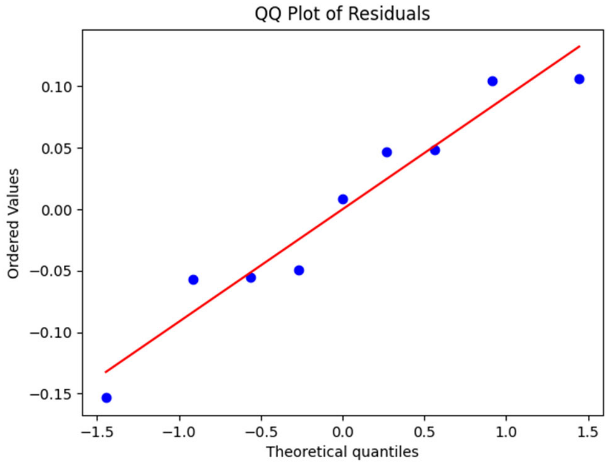

Figure S14. Q-Q plot of residuals for Friedelin concentration data.

As the p-value of the Shapiro-Wilk test (p=0.5114) was greater than the significance threshold of 0.05, the null hypothesis (that the data comes from a normal distribution) was not rejected. This indicated that the residuals were consistent with a normal distribution. Furthermore, the Q-Q plot (Figure S14) showed that the points lay close to the diagonal line, visually confirming the normal distribution of residuals and satisfying this assumption.

1.2. Homogeneity of variances:

Due to the nature of this dataset (one value per solvent-temperature combination, i.e., no replication), Levene's test could not be directly applied to assess homogeneity of variances. Instead, a qualitative assessment was performed by graphing residual values against adjusted (fitted) values.

```
# 6. Residuals vs Fitted
plt.figure()
plt.scatter(df['Predicted'], df['Residual'])
plt.axhline(y=0, color='gray', linestyle='--')
plt.xlabel('Fitted Values (Predicted Friedelin concentration)')
plt.ylabel('Residuals')
plt.title('Residuals vs Fitted Values')
plt.grid(True)
```

Figure S15. Python code for generating the residuals vs. fitted values plot for Friedelin concentration data.

The plot (Figure S16) showed that the residuals were scattered randomly around the zero line and were fairly balanced between positive and negative values. This visual inspection provided no strong evidence of heteroscedasticity, thus supporting the assumption of equal variances (homoscedasticity).

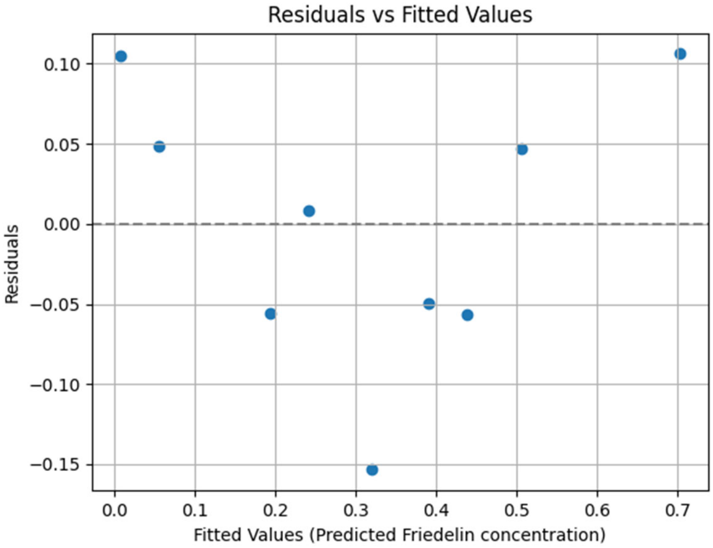

Figure S16. Residuals vs. fitted values plot for Friedelin concentration data.

1.3. Independence of residuals:

The independence of residuals was ensured by the experimental design and the methodology employed for data collection. Each extraction was performed separately for each condition, utilizing independent root samples in distinct Erlenmeyer flasks with fresh solvent and constant sample size/volume. This systematic approach minimized the potential for samples to influence one another. Additionally, visual inspection of the residuals vs. fitted values plot (Figure S16) further confirmed a random scatter of residuals, supporting the assumption of independence.

2. Antioxidant Activity Data

The following codes were applied for all antioxidant results data (RDSC values, TEAC values, and % of Inhibition).

2.1. Normality of residuals:

Normality of residuals was assessed using the Shapiro-Wilk test and a Q-Q plot.

```python
import pandas as pd
import numpy as np
import matplotlib.pyplot as plt
import scipy.stats as stats
import statsmodels.api as sm

# --- 1. Load data from Excel file
df = pd.read_excel("DPPH results.xlsx")

# --- 2. Melt to long format (for ANOVA with replication)
df_long = df.melt(id_vars=["Solvent", "Temp"], value_vars=["Trial 1", "Trial 2", "Trial 3"], var_name="Trial", value_name="RDSC")

# --- 3. Calculate group means (predicted values)
group_means = df_long.groupby(["Solvent", "Temp"])["RDSC"].transform("mean")

# --- 4. Calculate residuals
df_long["Residual"] = df_long["RDSC"] - group_means

# --- 5. QQ plot of residuals
sm.qqplot(df_long["Residual"], line="s")
plt.title("QQ Plot of Residuals (RDSC - Predicted RDSC)")
plt.show()

# --- 6. Shapiro-Wilk test for normality of residuals
shapiro_stat, shapiro_p = stats.shapiro(df_long["Residual"])
print(f"Shapiro-Wilk Test: Statistic = {shapiro_stat:.4f}, p-value = {shapiro_p:.4f}")
```

Figure S17. Python code for Shapiro-Wilk test and Q-Q plot generation for antioxidant activity data residuals.

Shapiro-Wilk Test results:

- RDSC values: Statistic = 0.9807, p-value = 0.5312
- TEAC values: Statistic = 0.9485, p-value = 0.1970
- % Inhibition values: Statistic = 0.9514, p-value = 0.2321

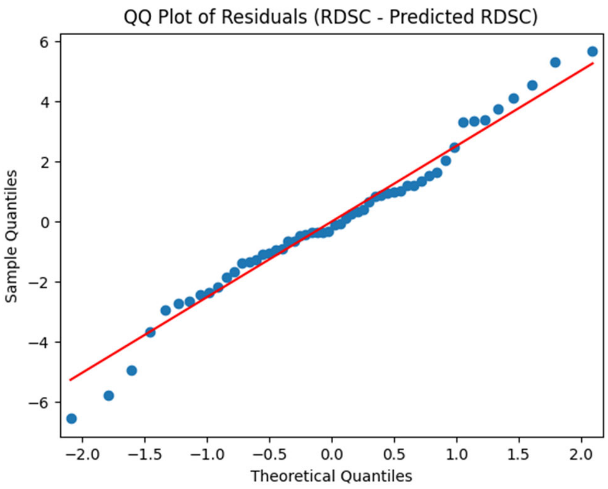

Figure S18. Q-Q plot of residuals for RDSC values

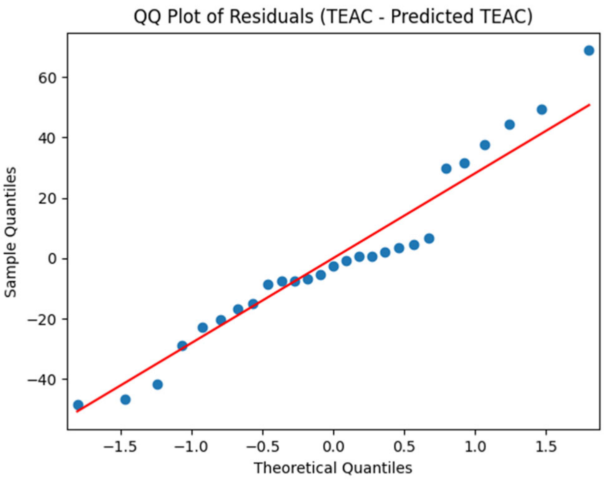

Figure S19. Q-Q plot of residuals for TEAC values

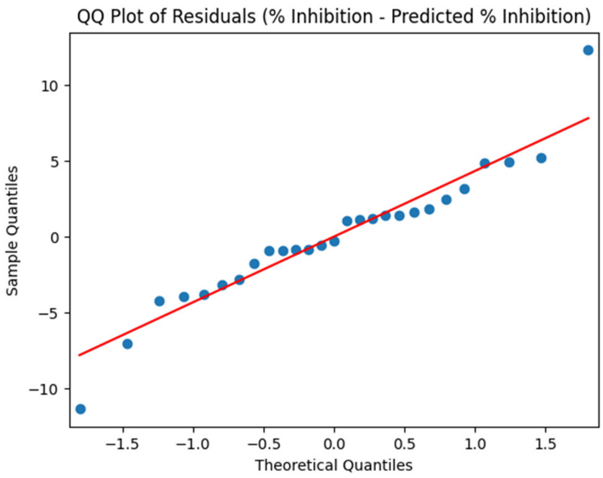

Figure S20. Q-Q plot of residuals for % Inhibition values

As all p-values of the Shapiro-Wilk test were greater than the significance threshold of 0.05, the null hypothesis was not rejected for all datasets, indicating that the residuals were consistent with a normal distribution. The Q-Q plots (Figure S18-S20) also confirmed that the points lay close to the diagonal line, satisfying this assumption.

2.2. Homogeneity of variances:

Homogeneity of variances was assessed using Levene's test and visually confirmed through a

```python
import pandas as pd
from scipy.stats import levene
import seaborn as sns
import matplotlib.pyplot as plt

# Load Excel file
file_path = 'DPPH results.xlsx'
df = pd.read_excel(file_path)

# Create a group label based on solvent and temperature
df['Group'] = df['Solvent'] + '_' + df['Temp'].astype(str)

# Reshape data from wide to long format for Levene's test
df_long = df.melt(id_vars=['Group'], value_vars=['Trial 1', 'Trial 2', 'Trial 3'], var_name='Trial', value_name='RDSC')

# Group data by Group and create a list of RDSC values for each group
grouped_data = [group['RDSC'].values for name, group in df_long.groupby('Group')]

# Perform Levene's test
stat, p_value = levene(*grouped_data)
print(f"Levene's Test Statistic = {stat:.4f}, p-value = {p_value:.4f}")

# Interpretation
if p_value > 0.05:
    print("✅ Variances are homogeneous (assumption met).")
else:
    print("⚠️ Variances are significantly different (homogeneity not met).")

# Sort groups for better visuals
df_long['Group'] = pd.Categorical(df_long['Group'], ordered=True, categories=sorted(df_long['Group'].unique()))
# Plot
plt.figure(figsize=(14, 6))
sns.boxplot(x='Group', y='RDSC', data=df_long)
plt.xticks(rotation=45)
plt.title('RDSC Variance by Solvent-Temperature Group')
plt.xlabel('Solvent_Temp Group')
plt.ylabel('RDSC Value')
plt.tight_layout()
plt.show()
```

Figure S21. Python code for Levene's test and boxplot generation for antioxidant activity data.

boxplot comparing the spread of antioxidant values across all solvent-temperature groups.

Levene's Test results:

- RDSC values: Statistic = 0.7844, p-value = 0.6981
- TEAC values: Statistic = 0.9222, p-value = 0.5213
- % Inhibition values: Statistic = 1.1854, p-value = 0.3601

Since the p-values of Levene's test were greater than 0.05, the null hypothesis (that all groups have equal variances) was not rejected, confirming homogeneity of variances across the groups. For the RDSC data, a visual inspection of the boxplot (Figure S22) showed that the spread (height of the boxes) across all groups did not differ dramatically, further supporting the assumption of equal variances. While some groups (particularly in MCT) exhibited slightly wider variability, it was not considered extreme enough to violate the assumption.

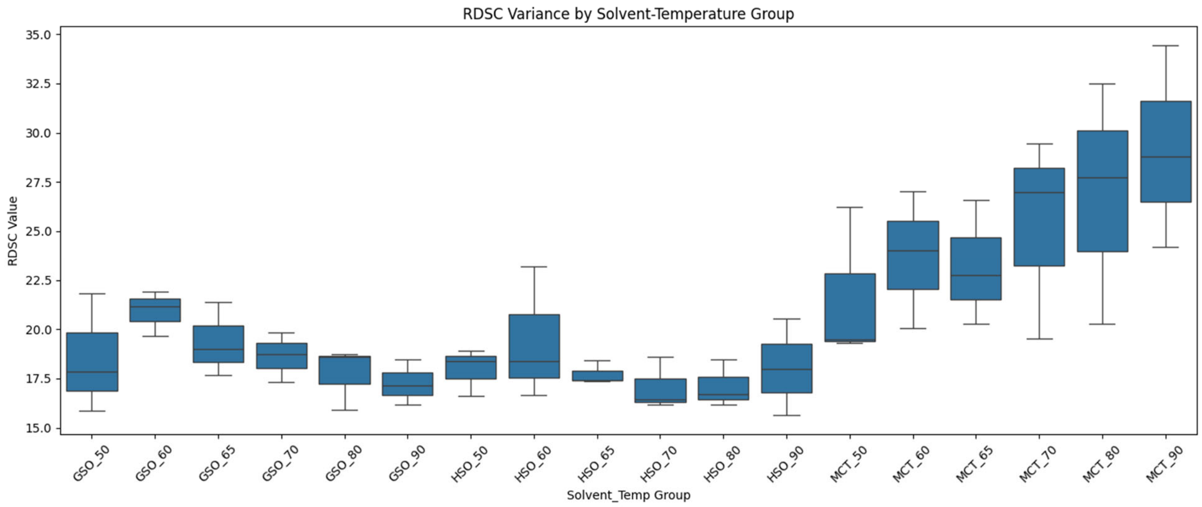

Figure S22. Boxplot showing variances of RDSC data across groups.

2.3. Independence of residuals:

The independence of residuals was ensured by the experimental design, where each antioxidant activity measurement was performed separately and independently for each condition. Additionally, a visual inspection was conducted by plotting residuals against fitted values.

```
import matplotlib.pyplot as plt
import seaborn as sns
import statsmodels.api as sm
from statsmodels.formula.api import ols
import pandas as pd

# Load data
df = pd.read_excel("DPPH results.xlsx")

# Convert factors
df['Solvent'] = df['Solvent'].astype('category')
df['Temperature'] = df['Temp'].astype('category')

# Melt the data into long format
df_melted = df.melt(id_vars=['Solvent', 'Temperature'], value_vars=['Trial 1', 'Trial 2', 'Trial 3'],
                    var_name='Trial', value_name='RDSC')

# Fit the model
model = ols('RDSC ~ C(Solvent) + C(Temperature) + C(Solvent):C(Temperature)', data=df_melted).fit()

# Residuals vs Fitted plot
plt.figure(figsize=(8,5))
sns.residplot(x=model.fittedvalues, y=model.resid, lowess=True, line_kws={'color': 'red', 'lw': 1}, scatter_kws={"alpha": 0.7})
plt.xlabel("Fitted RDSC Values")
plt.ylabel("Residuals")
plt.title("Residuals vs Fitted Values")
plt.grid(True)
plt.show()
```

Figure S23. Python code for plotting residuals vs. fitted values for antioxidant activity data.

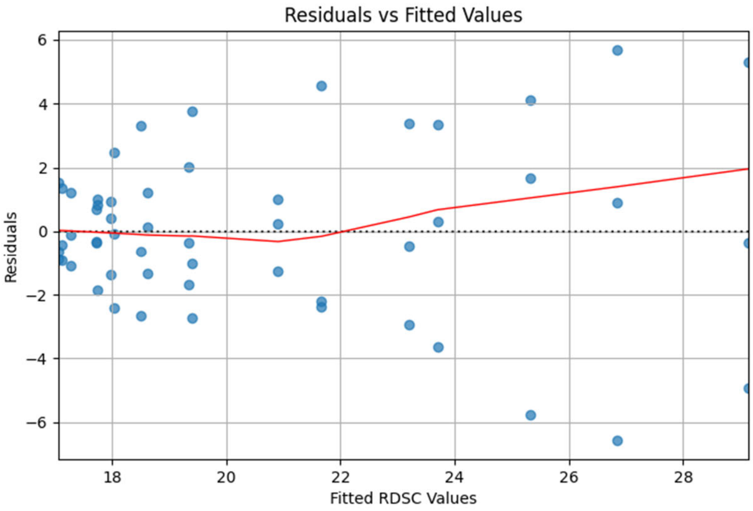

Figure S264. Residuals vs. fitted values plot for RDSC data.

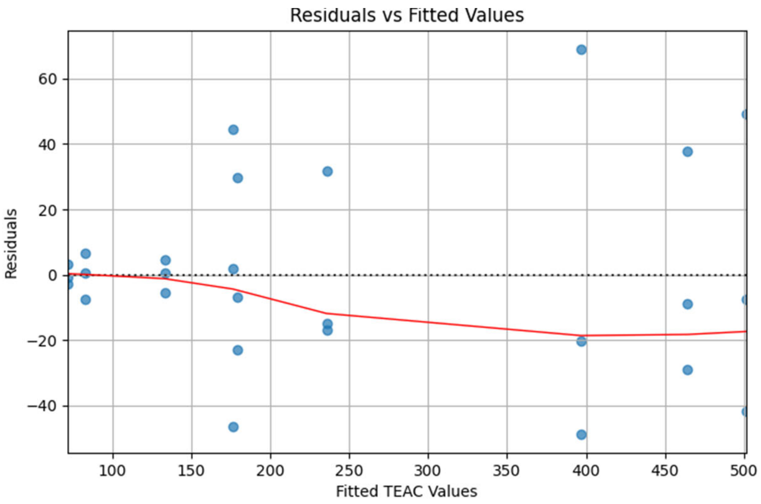

Figure S25. Residuals vs. fitted values plot for TEAC data.

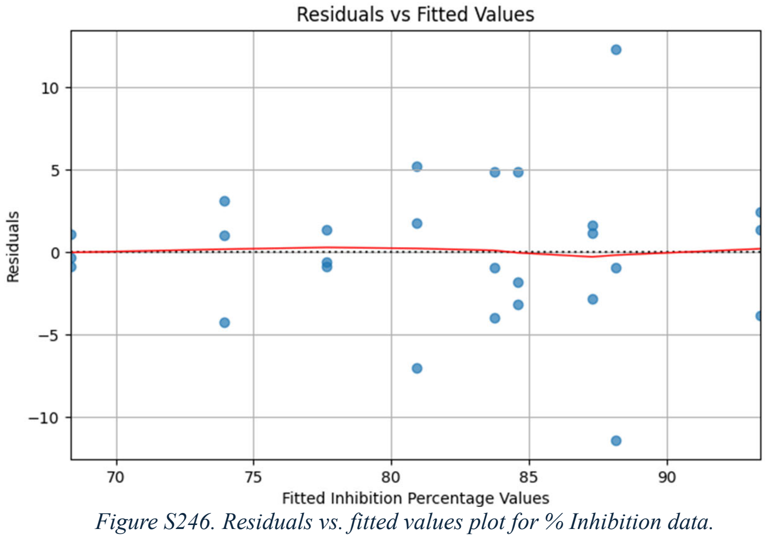

Figure S246. Residuals vs. fitted values plot for % Inhibition data.

The residuals vs. fitted values plots (Figure S24-S26) showed a fairly even scatter of residuals, with the LOWESS smoothing lines exhibiting no strong trend. This indicated that the residuals were approximately independent and randomly scattered around zero, confirming the satisfaction of this assumption.

D. ANOVA: Statistical Analysis Scripts and Results

All statistical analyses were performed using Python libraries such as statsmodels, pandas, numpy, and scipy. The following Python code snippets were used to conduct the Two-Way ANOVA analyses for both Friedelin concentration and antioxidant activity, including post-hoc Tukey's HSD tests.

1. Friedelin Concentration Data

For the Friedelin concentration data, a Two-Way ANOVA without replication was conducted

```python
# ========== TWO-WAY ANOVA WITHOUT REPLICATION ==========
# Step 1: Create a pivot table (Solvent = rows, Temperature = columns)
pivot = df.pivot(index='Solvent', columns='Temperature', values='Friedelin')
# Step 2: Calculate SS terms
grand_mean = pivot.values.mean()
a_levels = pivot.index
b_levels = pivot.columns
# Sum of squares for factor A (Solvent)
SSA = sum([(pivot.loc[i].mean() - grand_mean)**2 for i in a_levels]) * len(b_levels)
# Sum of squares for factor B (Temperature)
SSB = sum([(pivot[col].mean() - grand_mean)**2 for col in b_levels]) * len(a_levels)
# Sum of squares for error
SSE = sum([(pivot.loc[i, j] - pivot.loc[i].mean() - pivot[j].mean() + grand_mean)**2
           for i in a_levels for j in b_levels])
# Degrees of freedom
dfA = len(a_levels) - 1
dfB = len(b_levels) - 1
dfE = dfA * dfB
# Mean squares
MSA = SSA / dfA
MSB = SSB / dfB
MSE = SSE / dfE
# F-values
FA = MSA / MSE
FB = MSB / MSE
# p-values
pA = 1 - stats.f.cdf(FA, dfA, dfE)
pB = 1 - stats.f.cdf(FB, dfB, dfE)
# Print ANOVA Table
print("\nTwo-Way ANOVA Without Replication:")
print("Source\t\tSS\t\tDF\t\tMS\t\tF\t\tp-value")
print(f"Solvent\t\t{SSA:.2f}\t\t{dfA}\t\t{MSA:.2f}\t\t{FA:.2f}\t\t{pA:.4f}")
print(f"Temp\t\t{SSB:.2f}\t\t{dfB}\t\t{MSB:.2f}\t\t{FB:.2f}\t\t{pB:.4f}")
print(f"Error\t\t{SSE:.2f}\t\t{dfE}\t\t{MSE:.2f}")
```

Figure S27. Python code for Two-Way ANOVA without replication for Friedelin concentration data.

Table S1. Summary of Two-Way ANOVA without replication results, detailing the F-values, degrees of freedom, and p-values for the effects of solvent type and extraction temperature on Friedelin concentration (ppm) in *Cannabis sativa* L. root extracts.

Source	SS	DF	MS	F	p-value
Solvent	0.2205	2	0.1102	7.42	0.0448
Temp	0.1707	2	0.0854	5.76	0.0663
Error	0.0592	4	0.0148		

2. Antioxidant Activity Data

For the antioxidant activity data, a Two-Way ANOVA with replication was performed, for all three datasets: RDSC values, ABTS values and %Inhibition.

```python
import pandas as pd
import numpy as np
import statsmodels.api as sm
from statsmodels.formula.api import ols

# --- 1. Load data from Excel file
df = pd.read_excel("DPPH results.xlsx")

# 2. Reshape from wide to long format
df_long = df.melt(id_vars=['Solvent', 'Temp'],
                  value_vars=['Trial 1', 'Trial 2', 'Trial 3'],
                  var_name='Trial', value_name='Trolox')

# 3. Run Two-Way ANOVA with replication (interaction model)
model = ols('Trolox ~ C(Solvent) + C(Temp) + C(Solvent):C(Temp)', data=df_long).fit()
anova_table = sm.stats.anova_lm(model, typ=2)

# 4. Display ANOVA table
print("Two-Way ANOVA With Replication:")
print(anova_table)
```

Figure S28. Python code for Two-Way ANOVA with replication for antioxidant activity data.

Following the ANOVA, Post-hoc Tukey's HSD tests were conducted to determine specific pairwise differences between group means, particularly to explore the significant main effect of solvent type on RDSC values.

```python
from statsmodels.stats.multicomp import pairwise_tukeyhsd
import matplotlib.pyplot as plt

# Tukey HSD test on Solvent effect
tukey = pairwise_tukeyhsd(endog=df_long['Trolox'],     # Dependent variable
                          groups=df_long['Solvent'],   # Grouping variable (Solvent)
                          alpha=0.05)                  # Significance level

# Print summary
print(tukey.summary())
```

Figure S29. Python code for Post-hoc Tukey's HSD tests for the effect of solvent type on RDSC.

ANOVA results - RDSC values

Table S2. Summary of Two-Way ANOVA with replication results, detailing the F-values, degrees of freedom, and p-values for the individual and interactive effects of solvent type and extraction temperature on Relative DPPH Scavenging Capacity (RDSC) (μmol Trolox equivalent per liter) in *Cannabis sativa* L. root extracts.

Source	SS	DF	F	p-value
Solvent	539.54	2	28.42	3.94×10^{-8}
Temperature	27.89	5	0.59	0.7094
Interaction (Solvent×Temp)	117.74	10	1.24	0.2999
Residual (Error)	341.78	36	—	—

Table S3. Summary table presenting the results of the Post-hoc Tukey's HSD tests for solvent type on RDSC values

Group 1	Group 2	Mean diff	p-adj	lower	upper	Reject
GSO	HSO	-0.8471	0.6912	-3.3347	1.6404	FALSE
GSO	MCT	6.2415	0	3.754	8.7291	TRUE
HSO	MCT	7.0887	0	4.6011	9.5762	TRUE

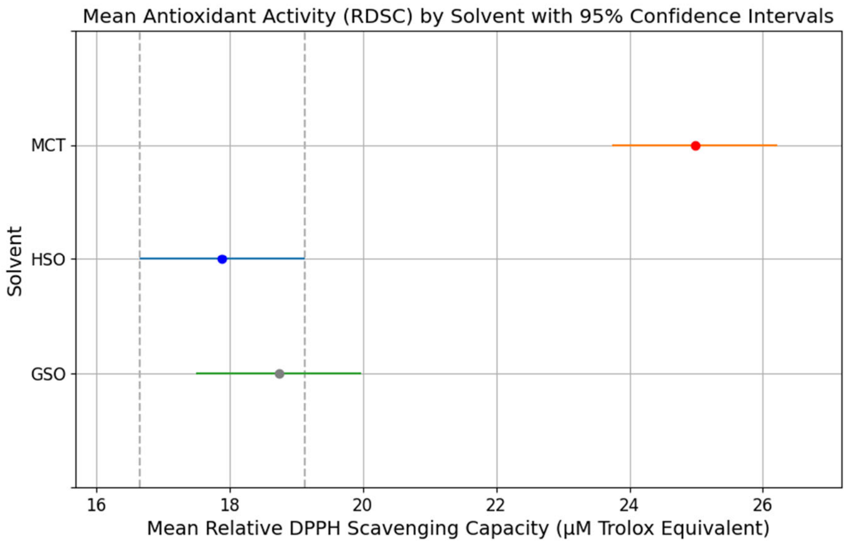

Figure S30. Tukey HSD Simultaneous Confidence Interval Plot for RDSC values

ANOVA results - TEAC values

Table S4. Summary of Two-Way ANOVA with replication results, detailing the F-values, degrees of freedom, and p-values for the individual and interactive effects of solvent type and extraction temperature on TEAC values, for ABTS test.

Source	SS	DF	F	p-value
Solvent	614886.20	2	258.92	5.44×10^{-14}
Temperature	12393.71	2	5.22	0.0163
Interaction (Solvent×Temp)	17664.77	4	3.72	0.0224
Residual (Error)	21373.44	18	—	—

ANOVA results - % Inhibition values

Table S5. Summary of Two-Way ANOVA with replication results, detailing the F-values, degrees of freedom, and p-values for the individual and interactive effects of solvent type and extraction temperature on % Inhibition values, for β-carotene bleaching method.

Source	SS	DF	F	p-value
Solvent	415.60	2	7.38	4.56×10^{-3}
Temperature	21.86	2	0.39	0.684
Interaction (Solvent×Temp)	992.33	4	8.81	4.00×10^{-4}
Residual (Error)	506.59	18	—	—

E. Uncertainty Analysis

The uncertainties associated with all reported experimental values were determined through standard error propagation methods. This approach accounts for the uncertainties in individual measurements and how they propagate through subsequent calculations.

General Formula for Error Propagation:

For a function f of independent variables $x_1, x_2, x_3, \ldots, x_N$ with associated uncertainties $\delta x_1, \delta x_2, \delta x_3, \ldots, \delta x_N$, the uncertainty in $\boldsymbol{f}$, denoted as δf, is calculated as:

$$f = f(x_1, x_2, x_3, \ldots, x_N) \tag{1}$$

$$\delta f = \left\{ \sum_{i=1}^{N} \left(\frac{\partial f}{\partial x_i} \cdot \delta x_i \right)^2 \right\}^{\frac{1}{2}} \tag{2}$$

Where $\frac{\partial f}{\partial x_i}$ represents the partial derivative of f with respect to x_i.

Uncertainty in Derived Quantities:

1. Uncertainty in % DPPH Quenched

The percentage of DPPH quenched was calculated using the following equation:

$$\%\,DPPH\ quenched_i = f(A_{sample,i}, A_{control,i}, A_{blank}) \tag{3}$$

$$\%\,DPPH\ quenched_i = \left(1 - \frac{A_{sample,i} - A_{blank}}{A_{control,i} - A_{blank}} \right) \times 100 \tag{4}$$

The uncertainty in % DPPH quenched ($\delta\%\,DPPH\ quenched_i$) was derived by propagating the uncertainties in the absorbance measurements ($A_{sample,i}, A_{control,i}, A_{blank}$. The specific equation used was:

$$\delta\%\,DPPH\ quenched_i = \sqrt{\left(\frac{\partial\%\,DPPH\ quenched_i}{\partial A_{sample,i}} \cdot \delta A_{sample,i}\right)^2 + \left(\frac{\partial\%\,DPPH\ quenched_i}{\partial A_{control,i}} \cdot \delta A_{control,i}\right)^2 + \left(\frac{\partial\%\,DPPH\ quenched_i}{\partial A_{blank}} \cdot \delta A_{blank}\right)^2} \tag{5}$$

Where:

$$\frac{\partial\%\,DPPH\ quenched_i}{\partial A_{sample,i}} = \frac{-100}{A_{control,i} - A_{blank}} \tag{6}$$

$$\frac{\partial\%\,DPPH\ quenched_i}{\partial A_{control,i}} = \frac{100(A_{sample,i} - A_{blank})}{\left(A_{control,i} - A_{blank}\right)^2} \tag{7}$$

$$\frac{\partial\%\,DPPH\ quenched_i}{\partial A_{blank}} = \frac{100(A_{control,i} - A_{sample,i})}{\left(A_{control,i} - A_{blank}\right)^2} \tag{8}$$

2. Uncertainty in Area Under the Curve (AUC)

The AUC was calculated using the following equation:

$$AUC = f(\%\, DPPH\ quenched_0, \ldots, \%\, DPPH\ quenched_{40}, \Delta t) \tag{9}$$

$$AUC = 0.5(\%\, DPPH\ quenched_0)(\Delta t) + \sum_{i=1}^{39} \%\, DPPH\ quenched_i(\Delta t) + 0.5(\%\, DPPH\ quenched_{40})(\Delta t) \tag{10}$$

Where $\Delta t = 1\ min$

The uncertainty in AUC (δAUC) was calculated as:

$$\delta AUC = \sqrt{\left(\frac{\partial AUC}{\partial \Delta t} \cdot \delta \Delta t\right)^2 + \sum_{i=0}^{40} \left(\frac{\partial AUC}{\partial \%\, DPPH\ quenched_i} \cdot \delta \%\, DPPH\ quenched_i\right)^2} \tag{11}$$

Where:

$$\frac{\partial AUC}{\partial \Delta t} = 0.5(\%\, DPPH\ quenched_0) + \sum_{i=1}^{39} \%\, DPPH\ quenched_i + 0.5(\%\, DPPH\ quenched_{40}) \tag{12}$$

$$\frac{\partial AUC}{\partial \%\, DPPH\ quenched_i} = \Delta t, \qquad for\ 1 \le i \le 39 \tag{13}$$

$$\frac{\partial AUC}{\partial \%\, DPPH\ quenched_i} = 0.5\ \Delta t, \qquad for\ i = 0{,}40 \tag{14}$$

3. Uncertainty in Net AUC

The Net AUC was calculated using the following equation:

$$Net\ AUC = f(AUC_{oil\ with\ extracts}, AUC_{oil\ without\ extracts}) \tag{15}$$

$$Net\ AUC = AUC_{oil\ with\ extracts} - AUC_{oil\ without\ extracts} \tag{16}$$

The uncertainty in Net AUC (δ Net AUC) was calculated as:

$$\delta\ \text{Net AUC} = \sqrt{\left(\frac{\partial\ NetAUC}{\partial AUC_{oil\ with\ extracts}} \cdot \delta AUC_{oil\ with\ extracts}\right)^2 + \left(\frac{\partial\ NetAUC}{\partial AUC_{oil\ without\ extracts}} \cdot \delta AUC_{oil\ without\ extracts}\right)^2} \tag{17}$$

So:

$$\delta\ \text{Net AUC} = \sqrt{(\delta AUC_{oil\ with\ extracts})^2 + (-\delta AUC_{oil\ without\ extracts})^2} \tag{18}$$

4. Uncertainty in Relative DPPH Scavenging Capacity (RDSC)

The RDSC was calculated by relating the Net AUC of the sample to the Trolox standard curve:

$$Net\ AUC = m_{slope}[Trolox] + b_{y-int} \tag{19}$$

Where $RDSC = [Trolox]$. So, RDSC was calculated as:

$$RDSC = f\left(Net\ AUC, b_{y-int}, m_{slope}\right) \tag{20}$$

$$\mathrm{RDSC} = \frac{AUC - b_{y-int}}{m_{slope}} \tag{21}$$

The uncertainty in RDSC ($\delta RDSC$) was propagated from the uncertainty in Net AUC ($\delta\ Net\ AUC$) and the uncertainties from the Trolox standard curve's linear regression fit ($\delta m_{slope}, \delta b_{y-int}$). The derived equation was:

$$\delta RDSC = \sqrt{\left(\frac{\partial\ RDSC}{\partial Net\ AUC} \cdot \delta Net\ AUC\right)^2 + \left(\frac{\partial\ RDSC}{\partial b_{y-int}} \cdot \delta b_{y-int}\right)^2 + \left(\frac{\partial\ RDSC}{\partial m_{slope}} \cdot \delta m_{slope}\right)^2} \tag{22}$$

Where:

$$\frac{\partial\ RDSC}{\partial Net\ AUC} = \frac{1}{m_{slope}} \tag{23}$$

$$\frac{\partial\ RDSC}{\partial b_{y-int}} = \frac{-1}{m_{slope}} \tag{24}$$

$$\frac{\partial\ RDSC}{\partial m_{slope}} = \frac{b_{y-int} - AUC}{m_{slope}^2} \tag{25}$$

5. Uncertainty in Average RDSC

Average RDSC was calculated as:

$$Av\ RDSC = f(RDSC_1, RDSC_2, RDSC_3) \tag{26}$$

$$Av\ RDSC = \frac{RDSC_1 + RDSC_2 + RDSC_3}{3} \tag{27}$$

The uncertainty in Average RDSC ($\delta\ Av\ RDSC$) was calculated as:

$$\delta Av\ RDSC = \sqrt{\left(\frac{\partial Av\ RDSC}{\partial RDSC_1} \cdot \delta RDSC_1\right)^2 + \left(\frac{\partial Av\ RDSC}{\partial RDSC_2} \cdot \delta RDSC_2\right)^2 + \left(\frac{\partial Av\ RDSC}{\partial RDSC_3} \cdot \delta RDSC_3\right)^2} \tag{28}$$

Where:

$$\frac{\partial Av\ RDSC}{\partial RDSC_1} = \frac{\partial Av\ RDSC}{\partial RDSC_2} = \frac{\partial Av\ RDSC}{\partial RDSC_3} = \frac{1}{3} \tag{29}$$
